# Disease-suppressive mechanisms in contrasting potato-based strip-cropping systems

**DOI:** 10.1007/s10658-025-03073-6

**Published:** 2025-05-26

**Authors:** Zohralyn Homulle, Paola Cassiano, Slava Shevchuk, Niels P. R. Anten, Tjeerd Jan Stomph, Wopke van der Werf, Jacob C. Douma

**Affiliations:** Centre for Crop System Analysis, Wageningen University, Wageningen, 6700 AK The Netherlands

**Keywords:** Strip cropping, Intercropping, Potato late blight, Disease suppression, Disease-suppressive mechanisms

## Abstract

Intercropping has been shown to suppress diseases in many crop-pathogen combinations and could be a component of more sustainable integrated crop protection. While various disease-suppressive mechanisms have been proposed, it remains unclear how different companion species influence these mechanisms, and whether trade-offs or synergies exist between them. Field experiments were conducted in the Netherlands to study various disease-suppressive mechanisms affecting late blight epidemics in potato strip-cropped with contrasting companion crops (grass, faba bean, or maize). Strip cropping significantly altered the microclimate in the potato strip; relative humidity was lower in potato-grass than in the potato monoculture, whereas the humidity was increased in potato-maize, especially later in the season. Strip cropping with faba bean did not significantly change the microclimate. Furthermore, potato-maize intercropping received the lowest number of particles over the growing season (a proxy for incoming spores). Strip cropping had little to no effect on potato plant morphology or canopy structure. Grass as a companion created drier conditions in the neighbouring potato canopy making it less conducive for disease development, while maize formed a barrier for spore dispersal though it increased humidity later in the season. But the barrier strategy appears a less certain approach across growing seasons, as it relies on the companion crop reaching sufficient height before the epidemic begins, but the timing of the epidemic is unpredictable and may be very early. This study offers insights into how companion species with specific traits can assist disease control in strip cropping.

**Supplementary Information:**

The online version contains supplementary material available at 10.1007/s10658-025-03073-6.

## Introduction

Crops are frequently exposed to biotic and abiotic stresses, which may damage them. Biotic stresses may be caused by weeds, arthropod herbivores (insects, mites), nematodes, bacteria, fungi and oomycetes. For the past sixty years, chemical biocides have been a mainstay in pest control, but these substances have negative environmental side effects (Mahmood et al., 2016; Sánchez-Bayo, 2011; Tilman et al., 2001). Biocides also pose health risks due to occupational exposure or spillover of pesticides to residential areas (European Environment Agency, 2023; Navarro et al., 2023; Ottenbros et al., 2023). Additionally, development of resistance to biocides negatively affects their efficiency over time (Fones et al., 2020; Gould et al., 2018). Future cropping systems need to be more resilient against pests and diseases while using fewer, or at least different and less environmentally harmful, pesticides than is currently allowed (Bryson, 2022; European Commission, 2020). Therefore, there is a need to explore ecologically based control options that are sustainable in the long run. Intercropping (the practice of growing multiple crop species in the same field at the same time) could be an interesting component of integrated crop protection, as numerous studies have confirmed its disease-suppressive potential (Boudreau, 2013; Stomph et al., 2020; van der Werf & Bianchi, 2022).

Mechanisms contributing to disease suppression in intercrop systems include the companion species acting as a barrier for the dispersal of disease propagules (hereafter referred to as the barrier effect), an altered microclimate in the host canopy (microclimate effect), a resistance response in the host elicited by the presence of the companion crop (induced host resistance or host susceptibility), and altered plant morphology and canopy structure of the host due to interactions with the companion crop (morphological effect) (Boudreau, 2013). These mechanisms will be further introduced in the following sections. Additionally, in replacement-type intercrop systems, i.e. mixtures created by replacing plants of one crop species with those of another such that the relative plant density total stays constant (van der Werf et al., 2021), the density of the host crop is reduced, which may, in turn, reduce the chance that a given pathogenic propagule reaches a host crop (dilution effect) (Boudreau, 2013; Hiddink et al., 2010). Although these mechanisms have been hypothesised and studied individually, it is largely unknown whether and how the mechanisms can be influenced by the identity and traits of the companion species, and how the effects of different mechanisms work out in combination (i.e., there being trade-offs or synergies). Such knowledge, though, could help our understanding of why certain crop combinations are more effective at disease suppression than others, and could thus improve intercrop designs to enhance disease management.

The companion crop's structure can influence the above-mentioned mechanisms. For example, Shtaya et al. (2021) found a negative correlation between faba bean rust severity and the height and fresh biomass of the accompanying cereal crop (oat, barley, wheat or triticale), indicating that the barrier's effectiveness depends on the height and likely the canopy density of the companion crop. Furthermore, the traits of companion crop species can affect the microclimate in the host canopy. A tall companion crop species will shade and reduce incoming radiation, resulting in a cooler canopy during the day, but potentially a warmer canopy during the night (Castro et al., 1991; Zhang et al., 2008). A tall and dense companion crop species can also influence the humidity in the host canopy by reducing wind speed and air movement, which decreases evaporation and increases relative humidity (Boudreau, 1993; Ong et al., 1991). In contrast, intercropping with a short companion crop creates a more open canopy with greater incoming radiation, and greater air movement, thereby often reducing relative humidity (Gómez-Rodríguez et al., 2003).

The presence of a companion crop can also induce morphological and physiological changes in the host plant, which could impact disease dynamics. For example, shading of a taller companion could cause the neighbouring host plants to elongate (Roig-Villanova & Martínez-García, 2016; Smith & Whitelam, 1997), which in turn can increase the host canopy porosity, affecting the microclimate inside the host canopy, generating favourable or unfavourable conditions for pathogen development (Calonnec et al., 2013; Tivoli et al., 2013). Plants also respond to changing light conditions by adjusting leaf size, leaf angle, leaf thickness and leaf mass (Chitwood et al., 2012; Dong et al., 2024; Ratjen & Kage, 2013; Wu et al., 2017). These anatomical features of leaves can affect the susceptibility of plants to disease (Ahn et al., 2020; Alonso-Villaverde et al., 2011; Smith et al., 2018). Furthermore, a low ratio of red to far-red light has been reported to decrease plants’ defence responses against pathogens, thereby increasing plant susceptibility (Cerrudo et al., 2012; de Wit et al., 2013). Lastly, incompatible pathogens or pollen dispersing from a companion species could elicit a resistance response in the host plant, protecting it against infection by virulent pathotypes (Finckh et al., 2000; Mundt, 2002).

The overall disease-suppressive effect of a certain companion crop species thus depends on the strength and direction of its influence on these mechanisms and the potential interactions among them. Indeed, strip cropping potato with grass, faba bean, or maize, resulted in varying levels of late blight suppression among the three companion crops (Homulle et al., 2024). On average over multiple years, strip cropping potato with different companion species reduced late blight by different percentages, e.g. 51% reduction with grass, 41% with maize %, and 22% with faba bean, when compared with potatoes grown in monoculture (Homulle et al., 2024). Since late blight (*Phytophthora infestans*) sporangia are mainly dispersed by wind (Aylor et al., 2001; Harrison & Lowe, 1989), a tall companion crop species was expected to act as a barrier for incoming spores, thereby reducing the incoming inoculum (He et al., 2010). A short companion crop species, on the other hand, could increase wind speed and solar radiation in the potato canopy, which would make the microclimate dryer, and less conducive for late blight development, thereby slowing down the progression of the epidemic (Bouws & Finckh, 2008; Ditzler et al., 2021). Furthermore, the different companion crop species could influence the morphology and susceptibility of the neighbouring potato plant and the structure of the potato canopy in various ways. Lastly, regardless of which companion crop species is used, each companion crop species occupies the space that would otherwise be occupied by potatoes, consequently lowering the number of hosts and the likelihood of a spore landing on a host.

The objective of this study was to investigate how these different companion crop species (grass, faba bean, maize) mediate disease-suppressive mechanisms. Potato was strip-cropped with either grass (shorter than potato), faba bean (slightly taller than potato at the time when late blight was present) or maize (considerably taller than potato at that time). We use potato and potato late blight as a focal crop-pathogen combination because *P. infestans* is considered to be the most devastating pathogen in potato (Campos & Ortiz, 2020; Majeed et al., 2017), and conventional growers rely heavily on fungicides to prevent and control potato late blight (Goffart et al., 2022; Yuen, 2021). Hence there is a great need to develop control options that are ecology based and sustainable in the long run.

## Materials and methods

In Homulle et al. (2024), we described disease suppression in the strip-crops using data from three experimental years (2021, 2022 and 2024), but we did not investigate the underlying mechanisms responsible for these results. We did in depth measurements on disease-suppressive mechanisms in one of the years (2022) and present the results here. Some of the measurements were also conducted during the other experimental year(s), details of these additional data, where available, are provided in Supplementary Information B.

### Field experiments

A field trial was conducted in 2022 at the organic experimental farm of Wageningen University & Research, located in Wageningen, The Netherlands. The trial was conducted at two experimental sites, located at approximately 850 m distance from each other (51°59′36"N 5°39′30"E (hereafter referred to as site A) and 51°59′32"N 5°40′16"E (site B), Fig. SA.1). Four experimental treatments were tested at each site: potato (*Solanum tuberosum* cv. Agria) grown in monoculture, potato strip-cropped with English ryegrass (*Lolium perenne*) (hereafter referred to as potato-grass), potato strip-cropped with faba bean (*Vicia faba* cv. Cartouche) (potato-faba bean), and potato strip-cropped with maize (*Zea mays*) (potato-maize). Two replicates of each treatment were at each site. As noted in “Data Analysis” section the data from this setup have a nested structure with plot nested within site (two sites with two treatment replicates per site). The potato cultivar used (Agria) is moderately susceptible in the foliage and fairly resistant in the tuber to potato late blight (The European Cultivated Potato Database, 2005). Potatoes were planted on 17 May 2022, faba bean was sown on 3 May, and grass and maize on 29 April 2022.

**Fig. 1:**
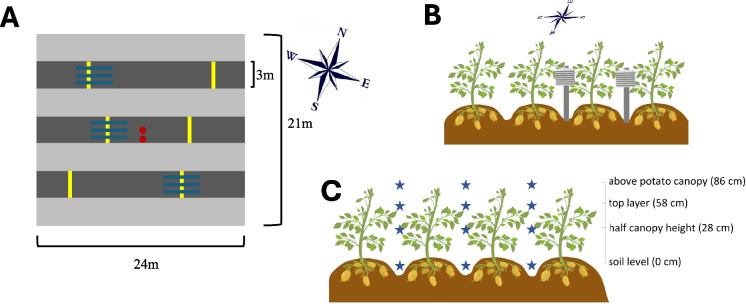
**A** Schematic arrangement of one experimental strip-cropping plot. Strips of each crop species were 3 m wide, and all strips were oriented from east to west. The plots had a size of 21 m × 24 m. The dark grey strips in the schematic arrangement represent potato, and the light grey strips either maize, grass or faba bean grown as a companion species. The yellow lines perpendicular to the strips represent transects for making disease assessments and measurements of crop height. Each transect consists of four plants, one per row. Transects were placed at random locations in each strip. Red dots indicate the position of the microclimate sensors. Blue lines parallel to the strip indicate where measurements of light interception were made with the SunScan (a 1-m long probe). **B** Side view of the placement of the microclimate sensors. Sensors were placed in the furrow between the potato rows. They were positioned roughly at the midpoint of the height of the canopy and the position was adjusted upwards throughout the growing season as the potato canopy grew in height. **C** Cross sectional view of the positions of the light interception measurements (the SunScan probe was directed parallel to the rows). Measurements with the Sunscan were made in three strips in each plot and per strip in three furrows and in each of these locations at four heights. The heights were: above the potato canopy (86 cm from the top of the soil), within the top layer of the potato canopy (58 cm), at half the potato canopy height (28 cm), and at soil level (0 cm)

Each plot measured 21 m in width × 24 m in length and comprised either only potato (monoculture plots) or alternating strips of potato and companion crops (strip-cropping plots). In strip-cropping plots, three 3 m wide potato strips were alternated with four 3 m wide strips of a companion species, such that the borders of the plots were strips of the companion species (Fig. 1). Each strip consisted of either four rows of potato (row width of 75 cm), four rows of maize, six rows of faba bean, or 20 rows of grass. The monoculture plots comprised 28 rows of potato at a 75 cm distance. In potato strips in strip-crops, a distinction was made between the inner rows that have only potato rows as direct neighbours and the outer rows that border on the companion species. Plots were separated by a 6 m strip of grass. Strips were planted in an east–west direction, corresponding to the prevailing wind direction in the Netherlands. The fields were managed organically; organic fertilizer was used and no pesticides or irrigation. For detailed information on crop management, see Homulle et al. (2024).

The summer of 2022 was warm and dry in comparison to the climatic mean for the area, with average daily temperatures around 18 °C during July, a relative air humidity level of 64%, and a total rainfall of 25 mm during July (Fig. SA.2).

### Disease assessment

During the growing season, foliar late blight severity caused by natural infections was assessed in all plots. We used two assessment methods: (1) counting the number of leaflets with lesions per plant, and (2) estimating the percentage diseased leaf area per plant. In the early stages of the epidemic, it was more accurate to count the number of leaflets with lesions per plant, rather than to estimate a very low percentage of diseased leaf area. As the epidemic progressed, counting the number of diseased leaflets was not possible anymore due to high disease severity, and only the percentage diseased leaf area per plant was recorded, following the classification scheme of James (1971). At some dates we conducted both measurements to enable calibration. We then used these data to perform a regression to convert the number of diseased leaflets into a percentage diseased leaf area (see Homulle et al. (2024) for details). This allowed for the combination of the two assessment methods into a single metric, hereafter referred to as disease severity.

To quantify disease severity, we randomly selected in each of the three potato strips in a plot two transects perpendicular to the strip, with each transect comprising four plants (Fig. 1A), resulting in a total sample of 24 plants per plot. In 2022, first symptoms were observed on 8 July, and assessments were made four times during the epidemic (on 12, 15, 19, 22 July at site A, and on 11, 14, 18, 21 July at site B). The plants were desiccated on 11 August 2022. The epidemic progress was halted around 19 July, when the weather was very hot with maximum temperatures reaching up to 36.6 °C, effectively killing all foliar lesions. After that, the epidemic did not progress much.

### Overview of the investigated mechanisms and their related measurements

**Fig. 2:**
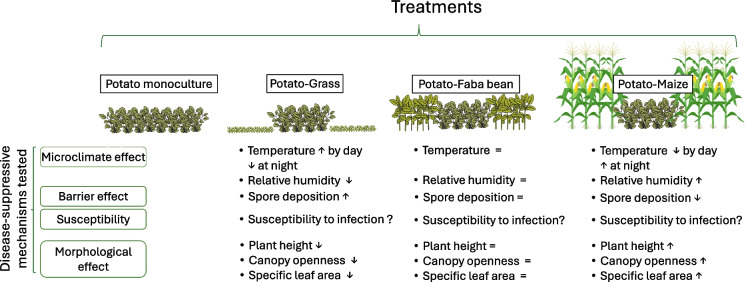
Overview of the treatments, the investigated disease-suppressive mechanisms, their related measurements and the hypothesised effects of the strip-cropping treatments compared to potato monoculture. The symbol ↑ indicates that the variable was hypothesized to be higher than in the monoculture, while ↓ signifies that it was expected to be lower. The symbol = is used when the variable was not anticipated to deviate significantly from the monoculture, and ? indicates uncertainty in forming a hypothesis. The canopy illustrations are for illustration only and do not accurately represent the actual height-to-width ratios

Within the treatments, various measurements were taken to investigate the relative role of different disease-suppressive mechanisms. See Fig. 2 for an overview of the hypothesized effects of strip cropping with different companion species.

### Microclimate measurements

Temperature and relative humidity inside the potato canopy were measured continuously over time in each of the four treatments, using MicroLite III Temperature/RH data loggers (LITE5032L-RH, Fourtec, Israel), which record both parameters. These data loggers were placed inside a radiation shield (model 7714, Davis Instruments, USA). The measurements were made in two plots per treatments at site A and in one plot at site B (hence in three out of the four replicates). In the strip-crops this was done in one strip per plot, placing one sensor in the furrow between the inner rows of the strip and the other one in the furrow between the third and fourth row, counted from the north side of the strip (Fig. 1A). In the monocultures, a single sensor was placed in a furrow in the middle of the plot. Sensors were positioned roughly at the midpoint of the canopy's height and were adjusted throughout the growing season as the potato canopy grew. All sensors were set to continuously record data every 10 min, starting from 16 June 2022 (early vegetative stage) until the end of the potato growing season (8 August 2022).

Wind speed and wind direction were measured continuously at the western (windward) edge of one experimental field with a single cup anemometer (6410 Davis Anemometer, Davis Instruments, USA). The anemometer was regularly adjusted to keep its measuring height level with the top of the potato canopy.

### Particle counts

To measure the role of the companion crop as a barrier for the dispersal of disease propagules, passive spore traps were used to catch particles slightly above the potato canopy. This was done over the whole growing season because the onset of late blight epidemics varies substantially and we wanted to assess barrier effect irrespective of the timing of the epidemic in our experiment. The assessment considered all particles in the size range of *P. infestans* sporangia. Additionally, the number of sporangia depends on the progression of the epidemic, which might differ between treatments because of the effect of the companion crop on microclimate and induced resistance. Thus measuring particles provides insight into what the barrier effect might be at any starting time of an epidemic, and independent of the epidemic.

To catch particles, we placed a passive spore trap in the middle of each replicate of each treatment. Passive spore traps were built using the design of Blackall et al. (2020) and Atkinson et al. (2019), see Supplementary method S1 for more details about the design of the passive spore traps. Three microscope slides, covered with a thin layer of Vaseline, were placed in each passive spore trap daily around 4 pm and collected the next day before 9 am for processing. To determine the density of particles deposited on the slide we took four pictures at 100 × magnification of each microscope slide using a microscope camera (ODC832, Kern and Sohn GMBH, Germany) in combination with the software Microscope VIS pro. Each photo represented an area of 12.57 mm^2^. We used ImageJ software to count particles within the size range of 314–1257μm^2^, i.e. approximately the size of *P. infestans* sporangia. This range was calculated based on the documented length and width of *P. infestans* sporangia (Mariette et al., 2018), and taking their elliptical shape into account.

### Detached leaf assays to measure susceptibility to infection

Companion species can affect the susceptibility of focal crops to pathogens, either due to induced resistance triggered by pollen or spores originating from companion plants, or due to other potential mechanisms such as nutrient competition, or volatile organic compounds. To measure the net effect of these influences on the susceptibility of potato, we performed a detached leaf assay (Lapwood, 1961; Vleeshouwers et al., 1999). We collected leaflets for this assay in the field, five weeks after potato planting (vegetative growth stage). We collected seven lateral leaflets from seven monocropped potato plants (one leaflet per plant), as well as seven leaflets from the inner rows and seven from both outer rows of strip-cropped potato plants. The youngest fully expanded leaflets were collected. The time of collection, five weeks after planting, was a compromise between allowing sufficient time for potato plants to interact with companion species and avoiding infection of the collected leaflets by naturally occurring sporangia.

Each leaflet was consequently placed upside-down on a water agar layer in a 100 mm Petri dish. The abaxial surface of each leaflet was inoculated with 10 droplets of 10 μL each, containing *P. infestans* sporangia (5000 sporangia/ml, strain EU_36). Five droplets were placed on each side of the midrib. Four leaflets per plot (two from the inner rows and two from the outer rows of the strip-crops, and two random leaflets of the monoculture plots) were inoculated with distilled water only and used as a control. All the samples were kept in a climate cell at 15 °C with 16 h of daylight. After five days, the number of lesions developed out of the 10 droplets was counted.

### Plant height

The height of the potato plants and each companion crop species was measured at four separate times during the growing season. At site A, measurements were taken at 32, 43, 49, and 67 days after planting (DAP), and at site B at 35, 42, 50, and 67 DAP. The measurements were made on 24 plants per plot, arranged in six transects of 4 plants each (same transects as for disease assessment, Fig. 1A). Height was measured from the potato ridge until the highest point of the potato plant. The height of 12 companion plants per plot (either grass, faba bean or maize) directly neighbouring the potatoes was measured as the distance between the soil surface and the highest point of the plant.

### Light interception

The openness of the potato canopy was characterized by measuring light interception at different heights of the potato canopy. The openness of the canopy determines the probability of a spore to be intercepted by the foliage. Measurements were taken once at eight weeks after planting. Again, this sampling date was a compromise between allowing sufficient time for the crop species to interact while avoiding a sampling date too late in the season, as late blight could reduce the canopy cover and thus influence the canopy openness. Photosynthetically active radiation (PAR) was measured in the potato canopies using a SunScan (SS1-STD3 by Delta T) (a 1-m long probe equipped with 64 individual sensors). Measurements were conducted from two hours before to two hours after solar noon. Light interception was measured at three locations in each potato monoculture plot and strip-plot (once per strip) (Fig. 1A). At each location, three positions were assessed: the probe was positioned parallel to the strip between the first and the second row counted from the north side of the strip, then between the second and the third row, i.e. in the middle of the strip, and finally between the third and fourth row of the strip. In the monoculture plots, per location, three positions in adjacent rows were assessed. Measurements were taken at four heights: above the potato canopy (86 cm from the top of the soil), within the top layer of the potato canopy (58 cm), at half the potato canopy height (28 cm), and soil level (0 cm) (Fig. 1C). Light interception was expressed as the ratio of PAR measured by the probe and PAR measured at the same time by a sensor placed at one-meter height in an unshaded point in the border of the field.

### Specific leaf area (SLA)

At 60 DAP, three lateral leaflets from separate leaves were collected per plant from 12 randomly selected plants per plot. The youngest fully expanded leaflets were collected. Leaf area was measured using a leaf area meter calibrated to mm^2^ (LI-3100 C by LI-COR Biosciences, Lincoln USA). Subsequently, the leaflets were dried in an oven at 105 °C for 24 h, and the dry weight was measured. Finally, SLA was calculated as the ratio of leaf area to leaf dry mass (cm^2^/g) and expressed as the average SLA of the three leaflets per plant.

### Data analysis

Differences in microclimate, particle count, susceptibility, and morphology aspects (plant height, canopy porosity and SLA) between treatments as well as differences between inner and outer rows of the strips within the strip-crops were analysed using (generalized) linear mixed models ((G)LMM). Mixed models were used to account for the nested structure of the data, with plot nested within site (two sites with two treatment replicates per site). In case measurements were done in different strips, strip was added as a third nested random effect, with strip nested in plot, and plot nested in site (models 5, 6, and 7, Table 1). For the variables measured over time (e.g. microclimate, particle count and plant height), day was included as a crossed random effect (models 1, 2, 3 and 5, Table 1). Models were fitted in R (R Core Team, 2022) using the package glmmTMB (Bolker, 2016; Magnusson et al., 2017).

**Table 1:** Summary of the fitted models to the data. The symbol + means additive effects are assumed, while * means main effects and their interactions are estimated. The notation A/B means B is nested in A

Model ID	Response variable	Distribution	Link function	Predictors	Random effects	Dispformula
1a	Mean daily temperature	Gaussian	-	Treatment	Day + Site/Plot	
1b	Temperature in either one of the strip-crops	Gaussian	-	Row position	Day + Plot	
2a	Daily duration (in min) relative humidity > 90%	Gaussian	-	Treatment	Day + Site/Plot	
2b	Daily duration (in min) relative humidity > 95%	Gaussian	-	Treatment	Day + Site/Plot	
2c	Daily duration relative humidity > 90% in either one of the strip-crops	Gaussian	-	Row position	Day + Plot	
3a	Particle count	Negative binomial ^a^	Logistic	Treatment + Slide	Day + Site/Trap/Slide	Treatment + Slide
3b	Particle count	Negative binomial ^a^	Logistic	Height companion crop + Slide	Day + Site/Trap/Slide	Height companion crop + Slide
3c	Particle count	Negative binomial ^a^	Logistic	Treatment * Wind Speed + Slide	Day + Site/Trap/Slide	Treatment + Slide
3 d	Particle count	Negative binomial ^a^	Logistic	Treatment * Day + Slide	Site/Trap/Slide	Treatment + Slide
4	*In vitro* infections	Binomial with zero inflation	Logit	Treatment	Site/Plot	-
5a	Potato plant height	Gaussian	-	Treatment	Day + Site/Plot/Strip	-
5b	Potato plant height in either one of the strip-crops	Gaussian	-	Row position	Day + Site/Plot/Strip	-
6a	Proportion of PAR captured	Beta	Logit	Treatment * Canopy level	Site/Plot/Strip	Treatment * Canopy level
6b	Proportion of PAR captured in either one of the strip-crops	Beta	Logit	Row position * Canopy level	Site/Plot/Strip	Canopy level
7a	Specific leaf area	Gaussian	-	Treatment	Site/Plot/Strip	-
7b	Specific leaf area in either one of the strip-crops	Gaussian	-	Row position	Site/Plot/Strip	-

*Treatment* is a categorical variable with four levels: monoculture, potato-grass, potato-maize or potato-faba. *Row position* represents the position of the rows within the strip, it has two levels: inner and outer (i.e. those in direct contact only with other potato plants, or with both potato and the companion crop). *Slide* represents the three microscope slides within one spore trap. *Canopy level* represents the height at which PAR was measured, and has four levels: above the canopy, in the top layer, in the middle layer and the bottom layer

^a^The negative binomial can be specified in two ways (NB-1 and NB-2). NB-2 performed better on the data than NB-1 (model 10 vs. model 11 in Table SA.1), therefore this distribution was used

Models for different response variables were tailored to fit the characteristics of the data (Table 1). If variables followed a normal distribution (e.g. temperature and plant height), we used LMMs; otherwise, we used GLMMs. Susceptibility (i.e. the 10 inoculation spots per leaflet of the detached leaf assay) was analysed using a binomial distribution (model 4, Table 1). Given the excess of zeros in the data set, the binomial distribution was enhanced to a zero-inflated binomial $(ziformula= \sim 1)$. The analysis of light interception used a beta distribution, since PAR captured was a proportion of total light (model 6a, 6b, Table 1). Since PAR was measured at multiple canopy levels, an interaction between treatment and height (categorical; 4 levels) was included in these models. Also, we accounted for heteroscedasticity across canopy height, using the *dispformula* argument of the glmmTMB function (Brooks et al., 2017).

The analysis of the particle counts used a negative binomial GLMM. Furthermore, initial exploration of the particle data showed that the top slides in the spore traps (Fig. SA.4) caught on average more particles than the middle ones, while the ones at the bottom captured the least. Therefore, slide position in the trap was added as a fixed factor within the models for particle data (models 3, Table 1).

In addition to comparing treatments, we also investigated the effect of companion crop height on incoming particle counts (model 3b, Table 1). A smooth curve was plotted through the weekly height measurements of the companion crops, from which the average daily height was obtained (Fig. SA.6). Lastly, wind speed and wind direction were tested as explanatory variables for particle count (model 3c Table 1, and models 1,2, 6 and 12 Table SA.1). As the microscope slides were present in the spore traps from approximately 16:00 until 9:00 the following day (when they were collected), the average wind speed during this time frame was considered. Wind direction was recalculated to a number between 0 and 1, to represent the perpendicularity of the wind direction in relation to the East–West orientation of the strip (see Supplementary methods S2). A value of one indicates that the wind was completely perpendicular to the direction of the strips (from the north or south), while a value of zero indicates that the wind was parallel to the strips (from the west or east). When wind speeds were zero, the anemometer would record 0° wind direction; these zeros were removed from the dataset.

## Results

### Disease severity

**Fig. 3:**
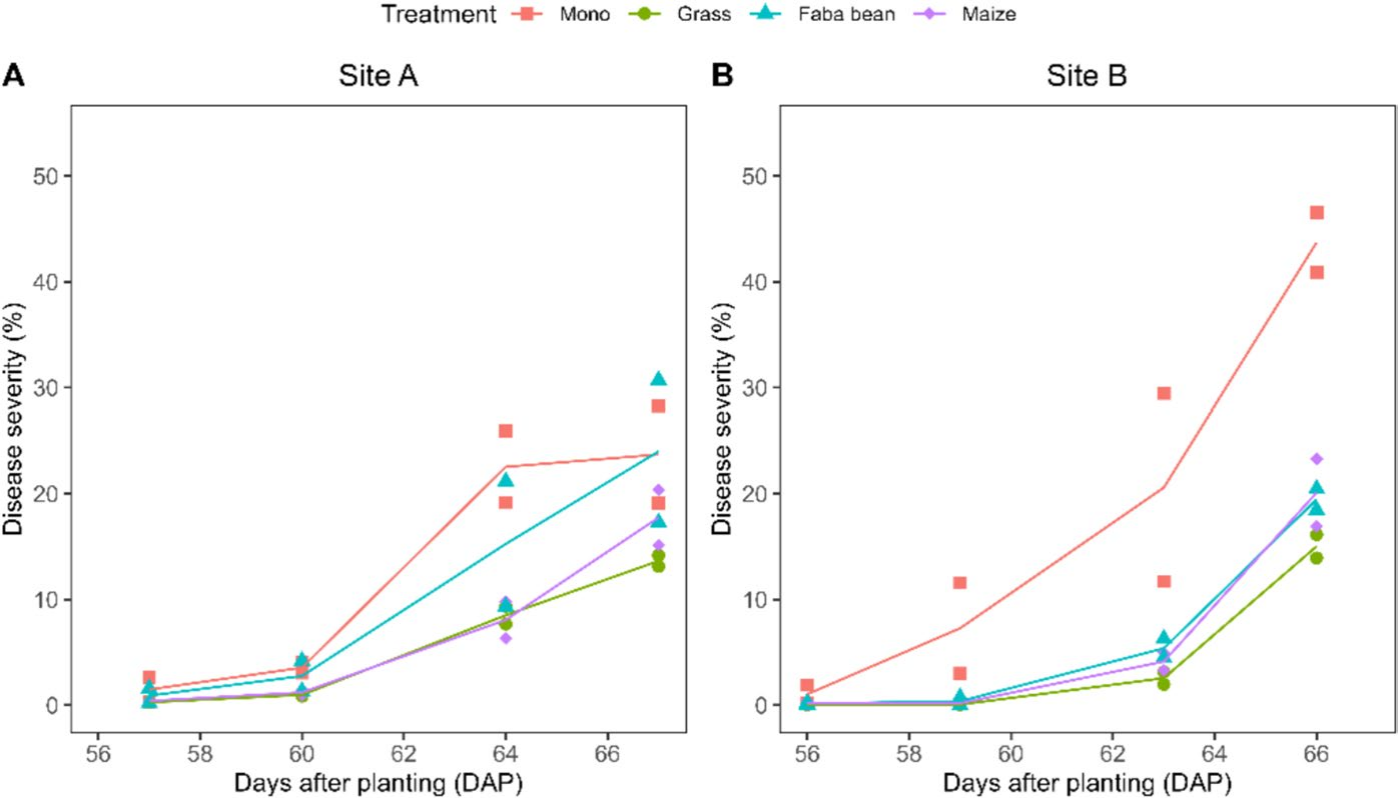
Disease progress curves for potato late blight on potato during the 2022 growing season at site A (**A**) and site B (**B**), modified from Homulle et al. (2024). The points (symbols) represent the mean disease severity per plot based on visual observations on 24 plants per plot. The lines are drawn between the midpoints of the two plots for each treatment

Average disease severity over the measurement period during the 2022 growing season was lowest in the potatoes strip-cropped with grass (5% (95% CI [4, 6]) at site A and 3% (95% CI [2, 4]) at site B), which was significantly lower than potato monoculture (11% (95% CI [8, 14]) at site A and 13% (95% CI [11, 17]) at site B) (Fig. 3A and B). Maize as a companion crop also significantly suppressed potato late blight compared with potato monoculture, with an average disease severity of 5% (95% CI [4, 7]) at site A and 4% (95% CI [3, 5]) at site B, estimates not significantly different from those of potato-grass, but significantly lower than sole potato. There was no significant difference in disease severity between the potato strip-cropped with faba bean and sole potato at site A (Fig. 3A). However, strip cropping with faba bean at site B suppressed potato late blight to a similar extent as maize did (average disease severity of 4% (95% CI [3, 6])) (Fig. 3B) and not significantly different from potato-grass.

Both the 2021 and 2024 growing seasons confirmed a consistent disease-suppressive effect of strip cropping potato with grass (Fig SB.2). The results for maize were more variable between years, and faba bean did not significantly suppress late blight in 2024.

### Microclimate in the potato canopy

We found no significant differences in temperature within the potato canopy between mono- and strip-cropped potatoes at any time during the growing season (Fig. SA.7). Both the average daily temperature and temperature variations during the day were similar and not significantly different (*p* > 0.9 in all comparisons). Furthermore, within the strip-crops, the temperature was similar in the inner and outer rows of the potato strip for all strip-cropping treatments.

We used the observed relative humidity within the potato canopy to assess whether and how many hours per day the microclimate in different treatments had been suitable for infection (RH ≥ 90% required) or sporulation (RH ≥ 95% required). We quantified the average number of hours per day with suitable conditions according to these two thresholds (Table 2) and found no significant differences between the treatments over the whole season. However, we observed significant differences when examining different periods in the growing season. From 1 to 10 July (the period surrounding the first finding of late blight in the field on July 8), the daily duration of relative humidity above 90% was similar in potato monoculture (11.5 ± 1.7 h), potato-maize (11.4 ± 2.0 h) and potato-faba bean (11.0 ± 2.4 h), but substantially and significantly lower in potato-grass (9.7 ± 1.9 h). Similar results were obtained for the duration of RH > 95%. Interestingly, towards the end of the growing season (25 July to 4 Augustus), the daily duration of relative humidity above 90% was significantly longer in the potatoes-maize (11.3 ± 3.8 h) than in potato monoculture (9.4 ± 3.6 h) (p = 0.03). Thus, the microclimate effects of strip cropping varied over the season, probably as the stature of the plants changed.

**Table 2:** Average daily hours with relative humidity equal to or exceeding 90% or 95%, and their respective standard deviations, throughout the full measurement period (from 16 June until August 7), around the time that late blight was first observed in the field (1–10 July)), and during the end of the growing season (25 July – 4 August, a period where maize was distinctly taller than potato). Letters indicate significant differences at *P* < 0.05 between treatments within each column

	16 June – 7 August		1–10 July		25 July – 4 August	
Treatment	RH ≥ 90%	RH ≥ 95%	RH ≥ 90%	RH ≥ 95%	RH ≥ 90%	RH ≥ 95%
Mono	9.8 ± 5.0a	7.2 ± 4.8a	11.5 ± 1.7a	8.8 ± 2.0a	9.4 ± 3.6a	6.5 ± 3.6ab
Potato-grass	9.1 ± 4.5a	6.5 ± 4.4a	9.7 ± 1.9b	7.0 ± 2.5b	9.1 ± 3.9a	6.3 ± 3.8a
Potato-faba bean	9.6 ± 5.0a	6.6 ± 4.8a	11.0 ± 2.4ab	7.4 ± 2.9ab	9.6 ± 4.3a	6.6 ± 4.2ab
Potato-maize	10.4 ± 4.7a	7.7 ± 4.7a	11.4 ± 2.0a	8.3 ± 2.7ab	11.3 ± 3.8b	8.6 ± 4.0b

Microclimate in the potato strip was also measured during the 2021 and 2024 growing season (SI B). During these years, temperature within the strip-crop potato canopy was also not influenced by the companion crop species (Fig SB.3). In 2021, around the period surrounding the first finding of late blight in the field, the daily duration of relative humidity above 90% was substantially lower in potato-grass than in the potato monoculture (Fig SB.4). This difference was not observed during the 2024 growing season. During this year, late blight arrived early when the potato plants were still young, and the growing season was considerably shorter than in 2021 and 2022, with potato plants desiccated on 9 July. These conditions could explain why no effects were observed.

**Fig. 4:**
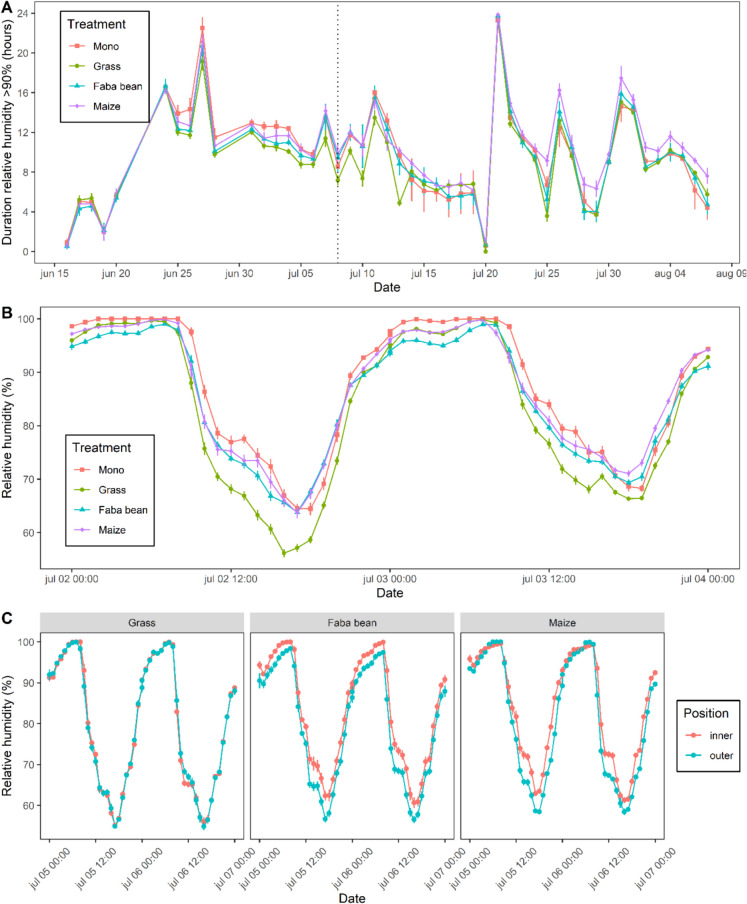
Relative humidity in the potato canopy for potatoes either grown in monoculture (Mono), or strip-cropped with grass, faba bean, or maize. **A** Daily hours with relative humidity equal to or exceeding 90% for each treatment across a part of the growing season. Vertical dotted line marks the first detection of late blight. **B** Hourly relative humidity for each treatment between 2 and 4 July; the time around which the first infections took place. **C** Hourly relative humidity of the inner and outer rows of potato strips in the strip-cropping treatments between 5 and 7 July

Furthermore, when considering the whole measurement period from 1 July to 4 August, in potato-maize and potato-faba bean, we found that the inner rows of the potato strip had a significantly longer duration with a high relative humidity than the outer rows (Fig. 4C). In potato-maize, the inner potato rows had an average duration of relative humidity above 90% of 10.9 ± 4.8 (SD) hours while the outer rows had a duration of 9.9 ± 4.6 (SD) hours (*p* < 0.01). In potato-faba bean, the inner potato rows had an average daily duration of relative humidity above 90% of 9.8 ± 4.8 h whereas the outer rows had a duration of 9.3 ± 5.1 h (*p* < 0.01). In potato-grass, there was no significant difference in relative humidity between the inner and outer potato rows (*p* = 0.55).

### Particle counts

**Fig. 5:**
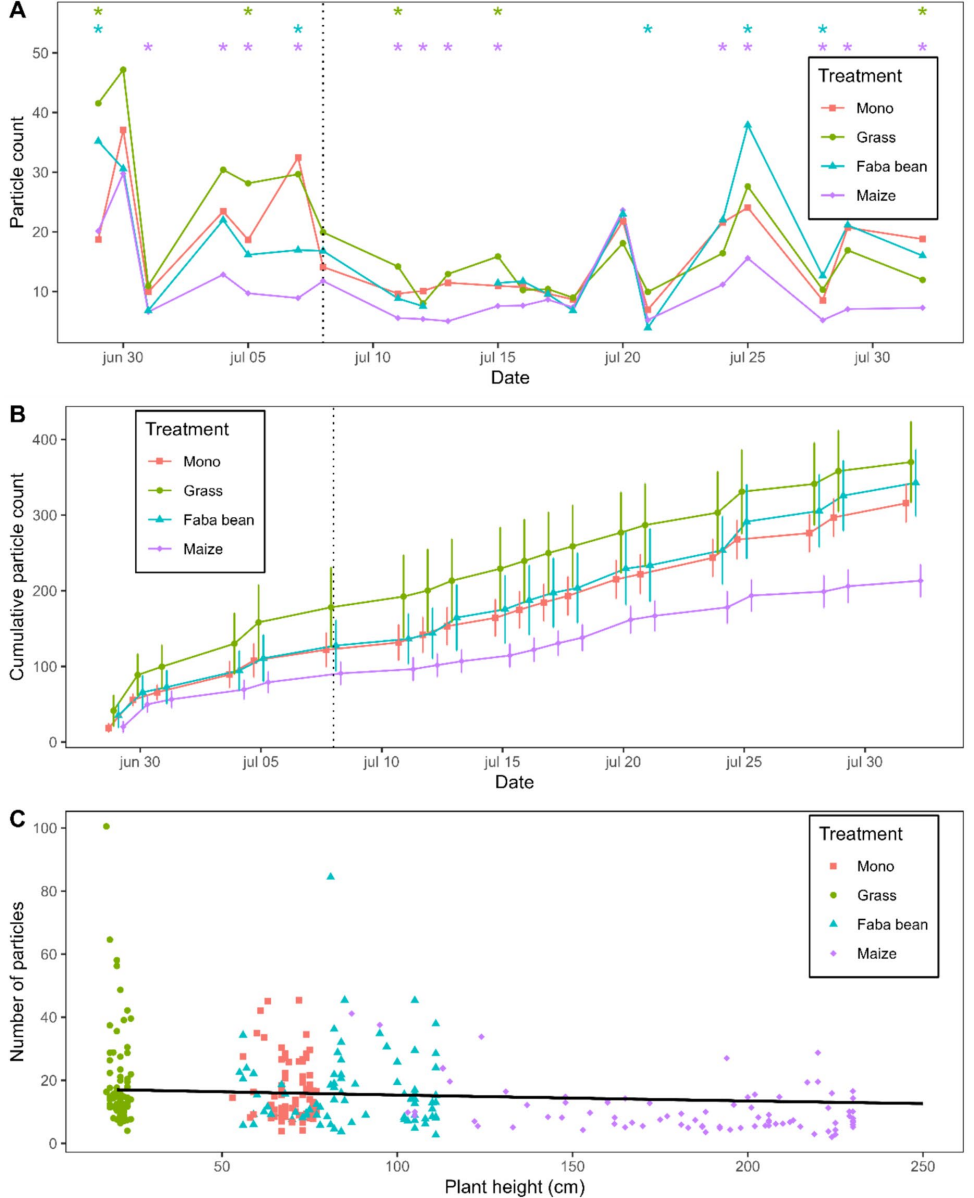
Particle counts in the potato canopy in potato mono (Mono), potato-grass, potato-faba, or potato-maize over the growing season, as an index for the barrier effect of the companion crop. Particles were sampled using Vaseline covered glass slides in passive spore traps placed in each plot. The vertical dotted line in A marks the first detection of late blight. **A** Particle counts on measurement days over the growing season. Stars indicate a significant difference between the strip-crop and the monoculture on a given day; top asterisk (green) for potato-grass, middle asterisk (blue) for potato-faba, and bottom asterisk (purple) for potato-maize. **B** Cumulative counts of measurement days across the season and their standard error. **C** Particle count in the potato canopy in relation to the height of the companion crop. Dots represent average particle counts per spore trap per day. The line represents the estimated regression: ${exp}^{2.86-0.0013x}$, where the slope is not significant (*p* = 0.063)

Potato-maize generally had the lowest number of particles in the size range of *P. infestans* sporangia out of all treatments, in line with the hypothesis that a tall companion species would act as a barrier against particle dispersal (Fig. 5A). On several induvial days, and across the growing season, this difference was significant. Summing the daily particle counts over the measurement period (30 June – August 1) showed an even clearer difference between the treatments (Fig. 5B). On average, across the growing season, potatoes strip-cropped with maize received in total 36% fewer particles than potatoes grown in monoculture (*p* < 0.001). The number of particles was not significantly lowered in potato-grass or potato-faba bean compared to potato.

There was weak support for a negative exponential relationship between the height of the companion crop and the number of intercepted particles (${exp}^{2.86-0.0013x}$, *p* = 0.063) (Fig. 5C). The height of the companion crop could, however, not predict particle count as effectively as the companion crop identity; the model using companion crop species as an explanatory variable (Table SA.1 Model 3) had a better fit to the data ($\Delta AIC= -48.4$) than a model using the height of the companion crop (Table SA.1 Model 9).

A significant interaction effect was found between treatment and wind speed just above the potato canopy (Chi-square test, χ^2^ = 28.86, df = 3, *p* < 0.001). Increasing wind speeds were associated with significantly lower particle counts in potato-maize or potato-faba bean than the monoculture (*p* < 0.001 for both comparisons) or potato-grass (grass-maize: *p* = 0.006; grass-bean: *p* = 0.09) (Fig. SA.8).

### Disease susceptibility

**Fig. 6:**
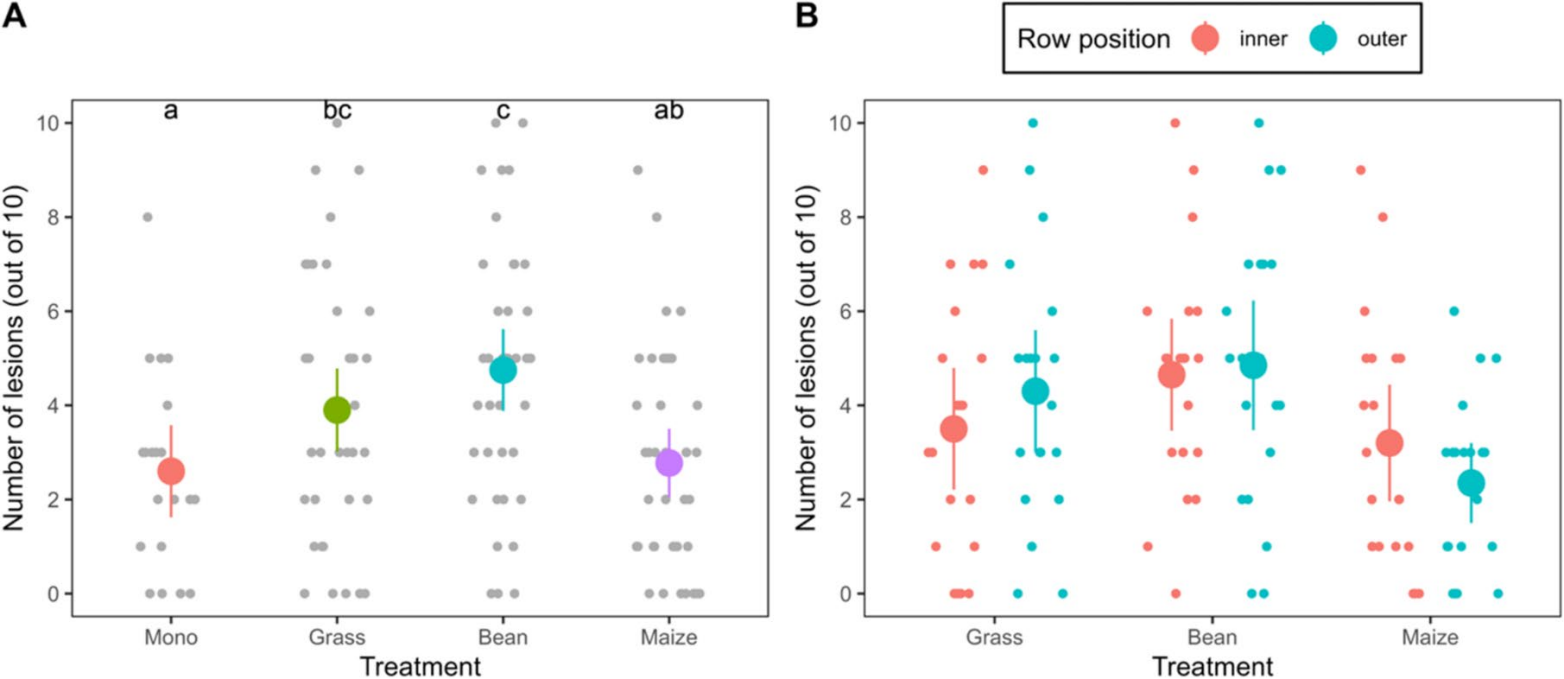
**A** Number of lesions out of 10 inoculations in detached leaf assays with leaflets from field-grown potato plants grown in monoculture (mono) or strip-cropped with grass, faba bean, or maize. Large circles represent the means and error bars indicate the confidence interval. The smaller points represent measurements on individual leaflets. Letters indicate significant differences between treatments at *P* < 0.05. **B** Number of lesions separately for inner and outer rows of potato strips in the strip-cropping treatments

Potato leaflets taken from the field and inoculated with *P. infestans* spores developed lesions (Fig. 6) while control samples (leaflets inoculated with water) did not develop any lesions, indicating that at the time of the detached leaf assay, 23 June, late blight was not present in the field, and all the lesions developed on the non-control samples resulted from the inoculation.

The average number of lesions that developed per treatment (out of 10 droplets) was 2.6 ± 0.5 in the monoculture, 2.8 ± 0.4 in potato-maize, 3.9 ± 0.4 in potato-grass and 4.8 ± 0.4 in potato-faba bean. There were significantly fewer lesions on leaflets from the monoculture than on those from potato-faba bean or potato-grass (*p* = 0.005 and 0.006, respectively), while no difference with potato-maize was observed (*p* = 0.6). In potato-grass or potato-faba bean, there was no significant difference in number of lesions between leaflets taken from the inner and outer rows. However, leaflets from the inner rows of potato-maize tended to develop more lesions than those from the outer rows (3.2 versus 2.3, *p* = 0.091).

A repeat of the detached leaf assay in a similar setup in 2024 showed no significant differences between treatments (Fig. SB.5).

### Structure of the potato canopy

**Fig. 7:**
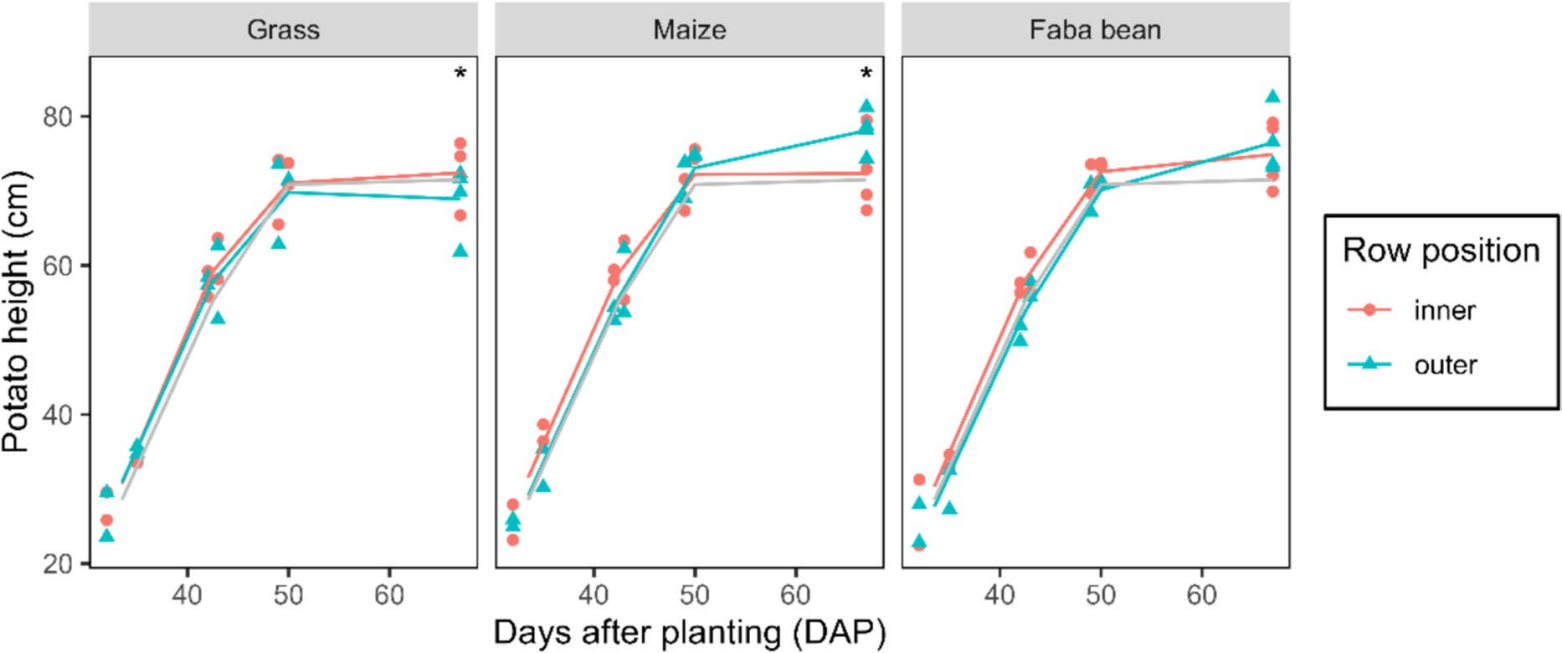
Height of potato plants in the inner and outer rows of potato-grass, potato-faba bean, and potato-maize across the growing season. Points represent the mean height of the potato plants in a plot, and lines represent the mean heights per row position. The grey line represent the average potato height in the monoculture for reference. Asterisks indicate significant difference between inner and outer rows (*p* < 0.05)

We found no significant difference in the height of the potato canopy between the strip-crops and the potato monoculture (Fig. SA.9). During the 2021 and 2024 growing season, also no height difference was observed between treatments (Fig. SB.7). However, within the potato strip, there were small differences in height between the inner rows of the strip and the outer rows (Fig. 7). These differences became only significant at the end of the growing season, at 67 DAP. Potatoes strip-cropped with grass were on average 3.5 cm taller in inner as compared to outer rows (*p* = 0.012) while potatoes strip-cropped with maize were on average 5.7 cm taller in outer as compared to inner rows (*p* = 0.014). These effects on plant height can be interpreted as shade avoidance responses, with plants growing taller when they have taller neighbours.

As expected, the proportion of total PAR captured decreased from the top to the bottom of the canopy (Table SA.3). At each canopy level, there were no significant differences among treatments indicating LAI and canopy porosity were not significantly affected by the treatments.

**Fig. 8:**
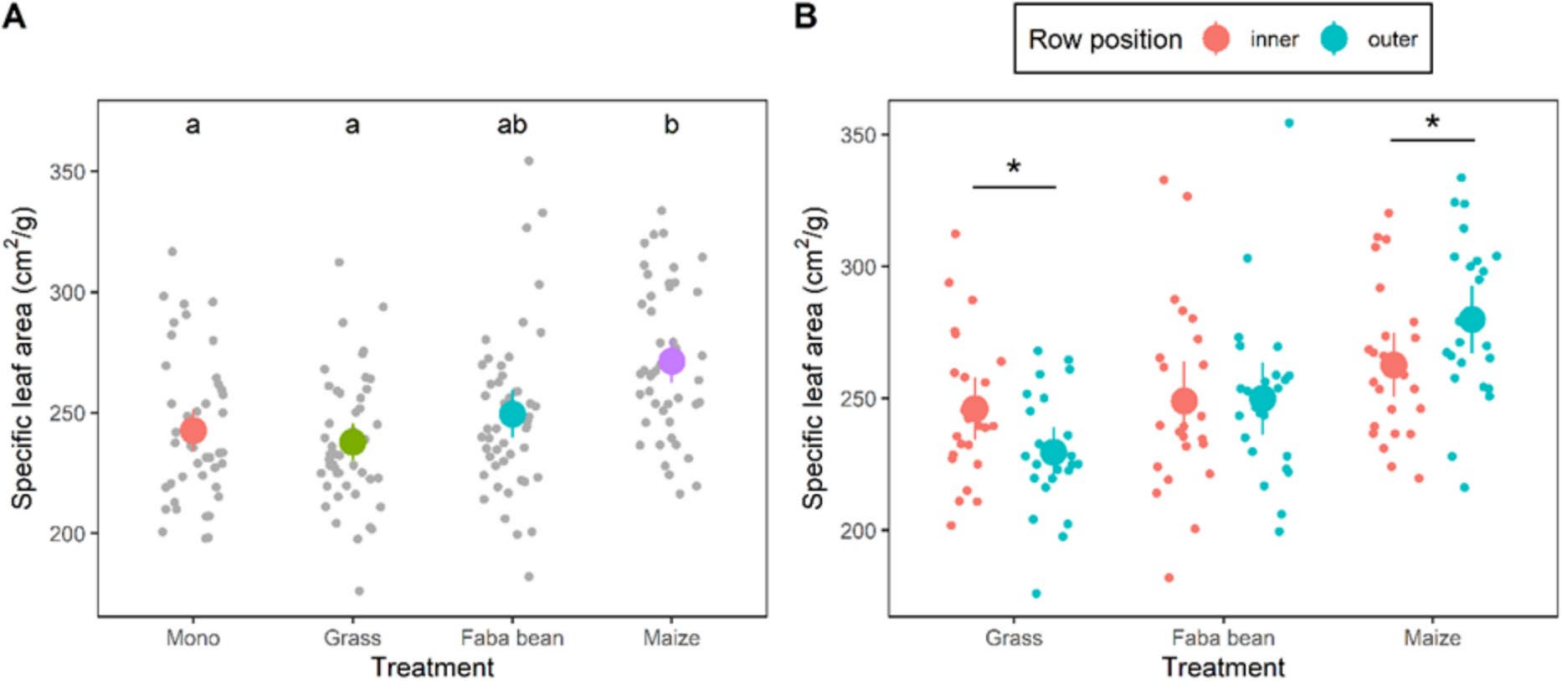
**A** Specific leaf area (cm^2^/g) from potato plants grown in monoculture (mono) or from potatoes strip-cropped with grass, faba bean, or maize. Large circles represent the means and error bars the confidence interval. The smaller points represent the average SLA per plant. Letters indicate significant differences between treatments at *P* < 0.05. **B** Specific leaf area of potatoes from inner and outer rows of the potato strips in the strip-cropping treatments. Asterisks indicate significant differences between inner and outer rows within a treatment at *P* < 0.05

Specific leaf area (SLA) of potato leaflets was significantly higher in potato-maize (271 ± 4.4 cm^2^/g) than in monoculture (243 ± 4.3 cm^2^/g), or potato-grass (238 ± 3.8 cm^2^/g) (*p* = 0.013 and *p* = 0.002, respectively) (Fig. 8A). Also, potato plants strip-cropped with maize had a significantly higher SLA in the outer rows compared with the inner rows (280 ± 6.2 cm^2^/g versus 263 ± 5.9 cm^2^/g, *p* = 0.018). The contrary was observed in potato-grass; here leaflets from the inner rows had a significantly higher SLA compared with the outer rows (246 ± 5.7 cm^2^/g versus 230 ± 4.6 cm^2^/g, *p* = 0.002) (Fig. 8B). As for plant height, these responses can be interpreted as shade adaptation as leaves that are well lit tend to be thicker, having a lower SLA, than leaves that are shaded.

## Discussion

In this study, we investigated how different identities and traits of companion crop species affect disease-suppressive mechanisms in potato-based strip-cropping systems. Disease suppression observed with each companion crop (Fig. 3) is achieved through different mechanisms. These mechanisms can either strengthen or counteract one another, resulting in potential synergies and trade-offs (see Table 3 for an overview of the outcome of the multiple mechanisms investigated in this paper).

**Table 3:** Summary of the investigated disease-suppressive mechanisms in the strip-crops

Mechanism	Key findings on each mechanism	Reference in text
Microclimate change	Measurements on microclimate in the potato canopy showed that a short companion crop (e.g. grass) decreases the duration of humid conditions that are conducive to infection and disease progress in the potato strip. A tall companion crop (e.g. maize) increased the relative humidity later in the growing season. We found no significant effect of strip cropping on temperature in the potato strip	Table 2, Fig, 4
Barrier effect on spore dispersal	Measurements on particle deposition showed that a tall companion species (e.g. maize) decreased deposition of particles, indicating that taller companion species would interfere with spore dispersal between potato strips	Figure 5
Change in susceptibility	Leaflets taken from potato-grass and potato-faba bean were slightly more susceptible to *in vitro* *P. infestans* inoculations than those from the monoculture	Figure 6
Change in morphology	Strip cropping with maize increased specific leaf area (SLA), particularly in the outer rows of the strip. In contrast, in potato-grass, the leaflets from the inner rows had a significantly higher SLA than the outer rows Potato canopy height and canopy openness showed no significant difference between strip-cropping and monoculture	Figure 8

Strip cropping with grass lowered the relative humidity in the potato canopy during the early epidemic, reducing the duration of favourable conditions for infection by almost two hours per day around the time of the first late blight detection. Changes in the duration of moist conditions are considered highly relevant for the epidemiology of potato late blight because daily patterns of humidity and leaf wetness duration (relative humidity > 90%, (Sentelhas et al., 2008)) impact several components of the pathogen’s lifecycle and relatively small differences in wetness duration can greatly affect disease progress. For instance, *P. infestans* sporangia are sensitive to drying (Minogue & Fry, 1981), so if the relative humidity decreases earlier during the day, their survival chance will be affected negatively. Likewise, sporangia are formed only if the humidity is at or very close to saturation (Harrison & Lowe, 1989), and infection requires a minimum of 2 to 3 h of leaf wetness to infect, but usually more, (Crosier, 1934), and a break in leaf wetness markedly reduces disease severity (Hartill et al., 1990). Humidity and wetness requirements were less often met in the potato-grass strip-crop than in the other systems and could thus have reduced the survival of sporangia and limited spore germination, thereby slowing down the onset and progress of the epidemic. Together this indicates that a short companion crop can make the conditions in the neighbouring host potato canopy less favourable for the late blight, though more testing with shorter companion crops other than grass is needed to verify the generality of this mechanism.

The positive drying effect of using a shorter companion crop (grass) was not associated with a significant increase in the number of particles arriving in the potato strip. However, using grass as a companion species influenced the susceptibility of the potato plants; leaflets taken from potato-grass were more susceptible to *in vitro* *P. infestans* inoculations than those from the monoculture. The short grass creates a more open canopy for the neighbouring potato strip, possibly exposing these potatoes to more mechanical stress from wind than those in monoculture. This stress can affect leaf traits, and has been reported to, among others, reduce leaf mass and leaf area (Anten et al., 2010). Interestingly, we observed that the outer potato rows of potato-grass had a significantly lower SLA than the inner rows, and the leaflets from these outer rows also tended to develop more lesions than the inner rows. In potato-maize a similar association between SLA and susceptibility was found; the outer rows had a significantly higher SLA compared with the inner rows, and leaflets from those outer rows tended to develop fewer lesions *in vitro* inoculation than leaflets from the inner rows. However, previous work on other species showed that leaves with low SLA are more resistant against fungal pathogens (O’Hara et al., 2016; Toome et al., 2010), possibly because these leaves are not infected as easily as thinner leaves (with high SLA). The response of detached leaves is, however, not always representative of attached leaves (Liu et al., 2007); and the detached leaf assay was only performed once during the season. A repetition of the detached leaf assay in 2024 did not confirm the current findings, stressing the need to investigate the reproducibility of these trends within and across seasons more closely.

Maize as a companion crop acted as a barrier for incoming particles to the neighbouring potato strip. Over the growing season, potatoes strip-cropped with maize received the lowest number of particles; we found an average reduction of 36% compared with potato monoculture. Such a reduction would logically translate to a proportional decrease in the incidence of primary infections caused by spores originating from outside the strip. Similarly, in an intercrop of pepper (*Capsicum annuum*) and maize, maize functioned as a physical barrier, lowering spore density within the pepper canopy, resulting in reduced anthracnose incidence (Gao et al., 2021). The height of the companion crop species likely influences how effective it will be as a barrier for spore dispersal. However, other aspects of the companion species, such as the leaf area density, are likely also important (Shtaya et al., 2021). This reasoning is supported by a model comparison that showed that the identity of the companion crop had greater explanatory power than the height of the companion crop alone. Although faba bean was slightly taller than potato, it did not provide an effective barrier, possibly due to its lower leaf area density (m^2^ leaf per m^3^ canopy volume) and earlier senescence (in late July) as compared to maize. In another intercrop experiment, while similar in height, barley, which produced more biomass than wheat, was more effective than wheat in reducing powdery mildew on pea (Villegas-Fernández et al., 2021).

Interestingly, within the potato-maize system, there was a trade-off between the barrier effect and the microclimate effect, and both effects varied over time but in opposite directions. The humidity increased in the potato strips next to maize, especially later in the season when maize was taller than potato and when late blight already had established. Dense crop canopies increase relative humidity and consequently the leaf wetness duration, due to reduced air circulation and slower leaf drying (Monteiro et al., 2006; Rowlandson et al., 2015; Vidal et al., 2017). While maize functions as a barrier, it may have thus in addition have created more conducive conditions to infection and epidemic progress.

Furthermore, the presence of maize also affected the morphology of the potatoes. We observed that the plants in the outer rows of the potato strip were taller than those in the inner rows; likely due to shading by the maize. Shading-induced elongation can influence plant and crop porosity, potentially affecting pathogen development (Calonnec et al., 2013; Tivoli et al., 2013). However, crop porosity (measured by light interception at different layers in the potato canopy) was not affected by strip cropping with maize, compared with the monoculture. It is therefore unlikely that strip-cropping induced changes in porosity, which subsequently could affect disease development.

Strip cropping with faba bean (slightly taller than potato) only slightly and not significantly lowered the duration of periods with high relative humidity in the potato canopy compared with potato monoculture (Table 2). Additionally, potatoes strip-cropped with faba bean received a similar number of particles as those in potato monoculture, and the morphology of potato plants was not significantly affected by faba bean. These findings suggest that the reduction in disease that was obtained by strip cropping with faba bean may have been due to a reduction in the area of potato, thereby resulting in spores landing on non-hosts which would tend to interfere with the rate of secondary spread. Similarly, simulation studies of spatially heterogeneous mixtures of susceptible and resistant potato plants show that as the number of susceptible units decreases, the probability of pathogen inoculum reaching another susceptible genotype also declines, as more inoculum is lost to non-hosts (Skelsey et al., 2005). In potato-grass, both the reduced potato area and the less conducive microclimate work together to enhance disease suppression, thereby leading to a higher disease suppression than in potato-faba bean. In potato-maize, while the barrier effect on spore dispersal is beneficial, the more humid microclimate may counteract these advantages, thus leading to a slightly lower disease suppression than in potato-grass.

### Practical implications

We measured various disease-suppressive mechanisms throughout one growing season. The strength of the various mechanisms will likely vary from year to year, depending on, for instance, the timing of the arrival of the first *P. infestans* spores in the field and the prevailing weather conditions during a certain year. For example, if the epidemic had started earlier in the season, maize would have been smaller in stature, which could have led to a reduced barrier effect against spore introduction. This was the case for the growing season of 2024 when late blight arrived early in the season, and maize was less effective in suppressing late blight than in the season reported here (Homulle et al., 2024). Hence, we speculate that strip-cropping potato with maize is a riskier disease-suppressive strategy compared to strip-cropping potato with a shorter companion crop, although the latter system might also fail in years with exceptionally high relative humidity. Indeed, grass-clover as a companion crop was more consistently effective than wheat in suppressing late blight (Bouws & Finckh, 2008). Although the strength of each mechanism may vary from year to year according to the prevailing weather and the time of onset of late blight epidemics, we expect the effect obtained by host dilution will always be present. Multi-year experiments would be required to test the consistency of mechanisms across growing seasons. Additionally, modelling might be an option to explore the relative effects of different factors, helping to simulate and predict outcomes of strip cropping under varying conditions.

Research is needed to explore how the disease suppressive effect varies with strip width. When strip cropping is done with wider strips, the conditions in the inner rows become more similar to monoculture (van Oort et al., 2020). For instance, in wider strips, the advantage of the less favourable microclimate in potato-grass could be diminished. Strip cropping potato with 6 m wide strips has indeed been found less effective in suppressing late blight than strip cropping with strips of 3 m (Ditzler et al., 2021). This observation aligns with the concept of Genotypic Unit Area (GUA), where increasing the area occupied by a single host genotype (increasing strip size) decreases interaction between species, leading to easier disease spread (Garrett & Mundt, 1999). In theory, strip widths smaller than 3 m would lead to stronger disease suppression. From the perspective of spores dispersing from within the potato canopy, it might be beneficial to have more intimate mixing of hosts and non-hosts, but turbulence and wind speed patterns will also change, making it hard to predict actual disease suppression. Strip width is thus an important factor affecting the disease-suppressive effect of strip cropping and requires careful consideration when designing strip-cropping systems. In such designs also labour costs related to management complexity and machine use will have to be considered.

## Conclusion

Various disease-suppressive mechanisms play a role in intercrop systems, and different companion crop species suppress disease through different (combinations of) mechanisms. Grass as a companion crop reduced the duration of humid conditions in the potato canopy, and it reduced late blight severity most out of the three different companion crops, suggesting that an unsuitable microclimate might be more important for suppressing late blight development than a reduction in incoming spores as the latter is partly countered by an increased humidity. A significant barrier effect was observed in the potato-maize, but the tall maize strips resulted in enhanced humidity in the strip-cropped potatoes, which counteracted the disease reducing barrier effect. Additionally, even if a companion crop species reduces the number of incoming spores, the few spores that manage to reach the potato canopy can start a late blight epidemic which can then spread quickly within the strips. Humidity, on the other hand, plays a major role at various stages in the disease cycle of potato late blight, and unsuitable conditions can thus slow down the progress of the epidemic. Moreover, since the barrier strategy depends on the companion crop reaching sufficient height before the epidemic begins and the time of arrival of the pathogen is hard to predict, it is presumably a less reliable disease suppressive strategy across different growing seasons. The findings in this paper provide useful clues on how the choice of companion species with specific traits can best assist in disease control by strip cropping.

## Supplementary Information

Below is the link to the electronic supplementary material.

Supplementary file1 (DOCX 7729 KB)

Supplementary file2 (DOCX 2611 KB)

## Data Availability

Data will be made available upon request.
