# Supplementary material for: Disease-suppressive mechanisms in contrasting potato-based strip-cropping systems
Source: Eur J Plant Pathol. 2025 May 26;173(2):469–89. doi: 10.1007/s10658-025-03073-6 (PMC12500844; doi:10.1007/s10658-025-03073-6)
Supplement: Supplementary file 1 — Supplementary file1 (DOCX 7729 KB) [file 10658_2025_3073_MOESM1_ESM.docx]

**Supplementary information A for: disease-suppressive mechanisms in contrasting potato-based strip-cropping systems**

Zohralyn Homulle^1^, Paola Cassiano^1^, Slava Shevchuk^1^, Niels P.R. Anten^1^, Tjeerd Jan Stomph^1^, Wopke van der Werf^1^, Jacob C. Douma^1^

^1^Centre for Crop System Analysis, Wageningen University, 6700 AK, Wageningen, the Netherlands

Corresponding author:

Zohralyn Homulle: zohralyn.homulle@wur.nl, [zohralyn@live.nl](mailto:zohralyn@live.nl)

**Table of Contents**

[Fig. SA.1. Map of the location of the two experimental sites. 2](#_Toc196731662)

[Fig. SA.2. Weather conditions during the 2022 growing seasons 2](#_Toc196731663)

[Method SA.1: Passive spore traps 3](#_Toc196731664)

[Fig. SA.3. Schematic drawing of a passive spore trap 4](#_Toc196731665)

[Fig. SA.4. Picture of the microscope slide holder inside the cylinder of the spore trap 4](#_Toc196731666)

[Method SA.2: From wind direction to a measure of perpendicularity 5](#_Toc196731667)

[Fig. SA.5. Schematic overview of the orientation of the strips 6](#_Toc196731668)

[Fig. SA.6. Height of the companion crops of the strip-crop treatments with grass, maize or faba bean, at site A and B. 7](#_Toc196731669)

[Table SA.1. Summary of the fitted models to the particle count data 8](#_Toc196731670)

[Fig. SA.7. Temperature in the potato canopy for potatoes either grown in monoculture, or strip-cropped with grass, maize or faba bean. 9](#_Toc196731671)

[Fig. SA.8. Particle count in the potato canopy in relation to the wind speed 10](#_Toc196731672)

[Fig. SA.9. Height of potato plants grown in monoculture, or strip-cropped with either grass, maize, or faba bean 10](#_Toc196731673)

[Table SA.3. Proportion of total PAR captured at four levels in the canopy of potatoes grown in monoculture or strip-cropped with grass, maize, or faba bean 11](#_Toc196731674)


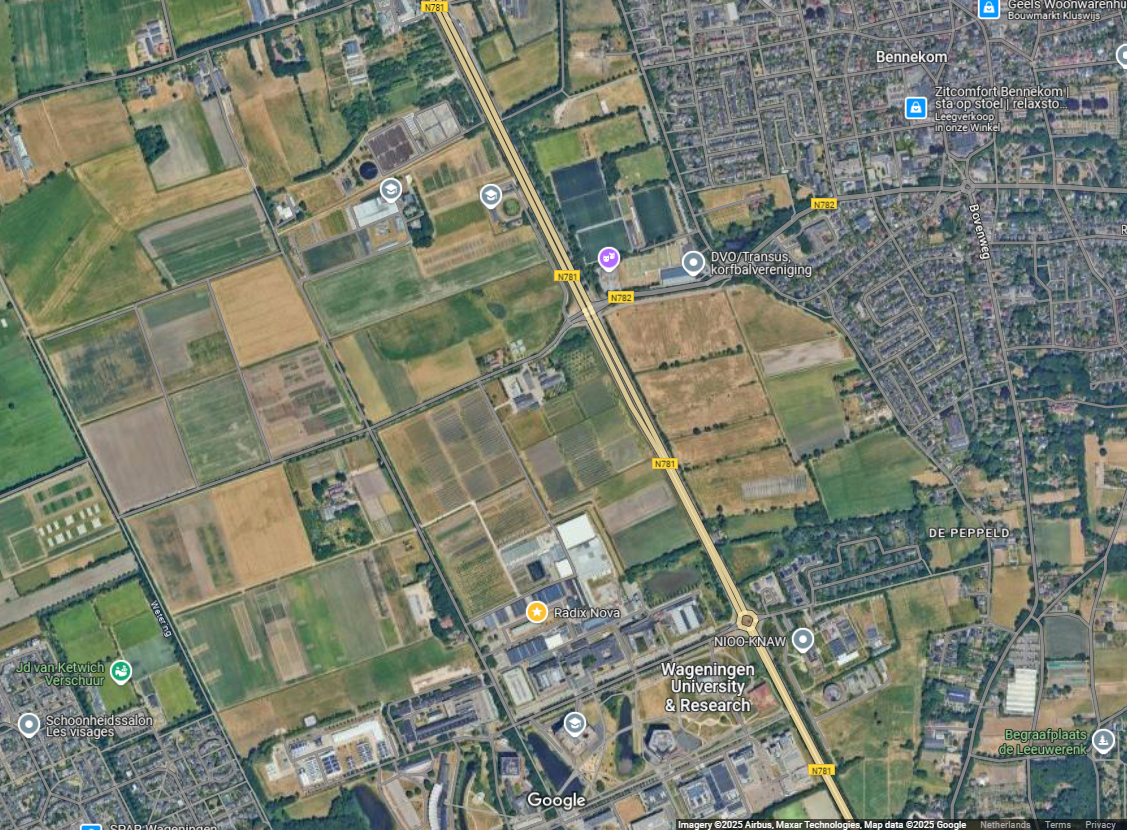

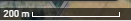


# Fig. SA.1. Map of the location of the experimental sites (red rectangles). The two experimental sites were located at approximately 850 meter distance from each other.


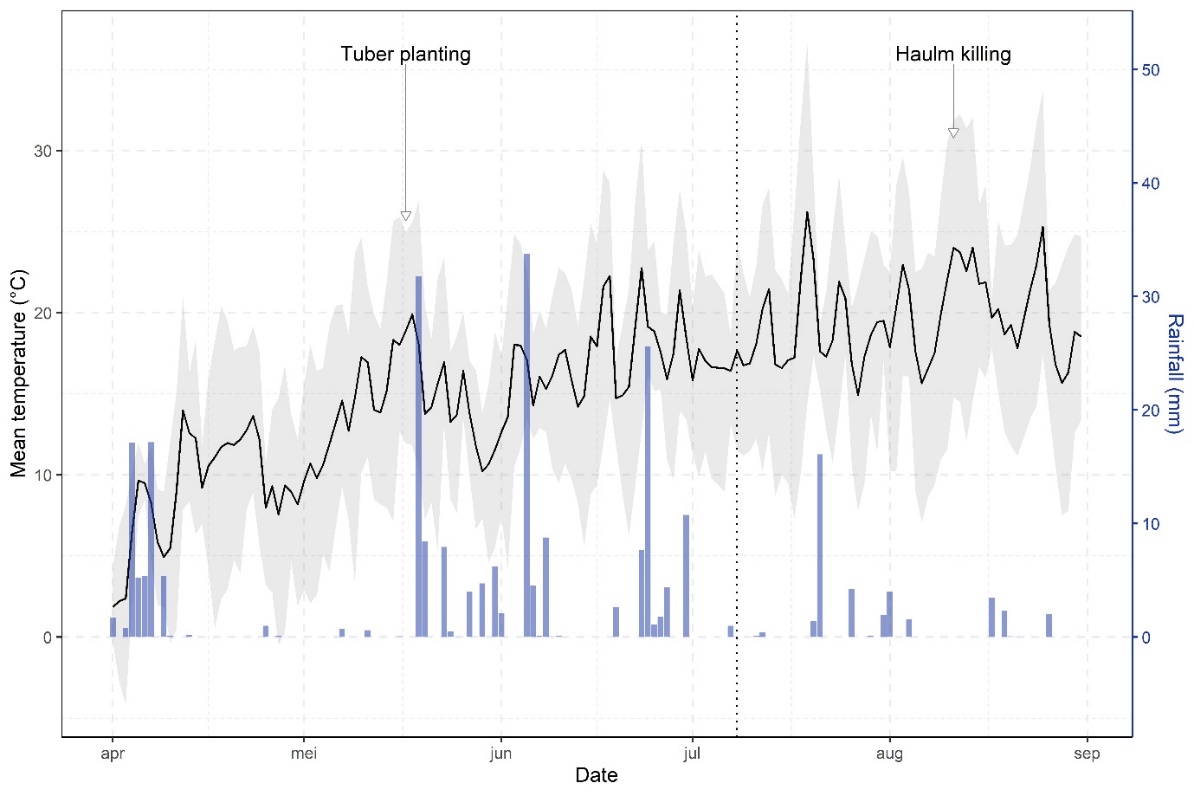


Fig. SA.2. Weather conditions during the 2022 growing seasons. The vertical dotted line mark the first detection of late blight. Black lines show mean temperature (degrees Celsius), grey ribbons span daily minimum and maximum temperatures, and blue bars are the total daily precipitation (mm). The dashes on the x-axis indicate the first of each month. Data was obtained from weather station De Veenkampen operated by Wageningen University, located approximately 3 km west of the experimental site.

# Method SA.1: Passive spore traps

Passive spore traps were designed to catch particles from the air onto greased microscope slides. The spore trap consists of a 33 cm long cylinder, with a 12.5 cm diameter, through which wind can pass. Attached to the cylinder is a trapezoid shaped fin, which ensures the movement of the cylinder to face into the prevailing wind (Fig SA.3). Both cylinder and fin were made of PVC. The cylinder was placed on a wooden pole of 150 cm, connected with a 20 cm long stud screw running throughout the cylinder. The stud screw was secured by a bolt and metal spacers at the top and the bottom of the cylinder, to ensure the cylinder was able to spin freely in the wind.

For within the cylinder, a microscope slide holder was made, which holds three microscope slides at a 45 degree angle (Fig SA.4). As wind passes across the surface of the greased slides, fine particles are captured on their sticky surfaces.


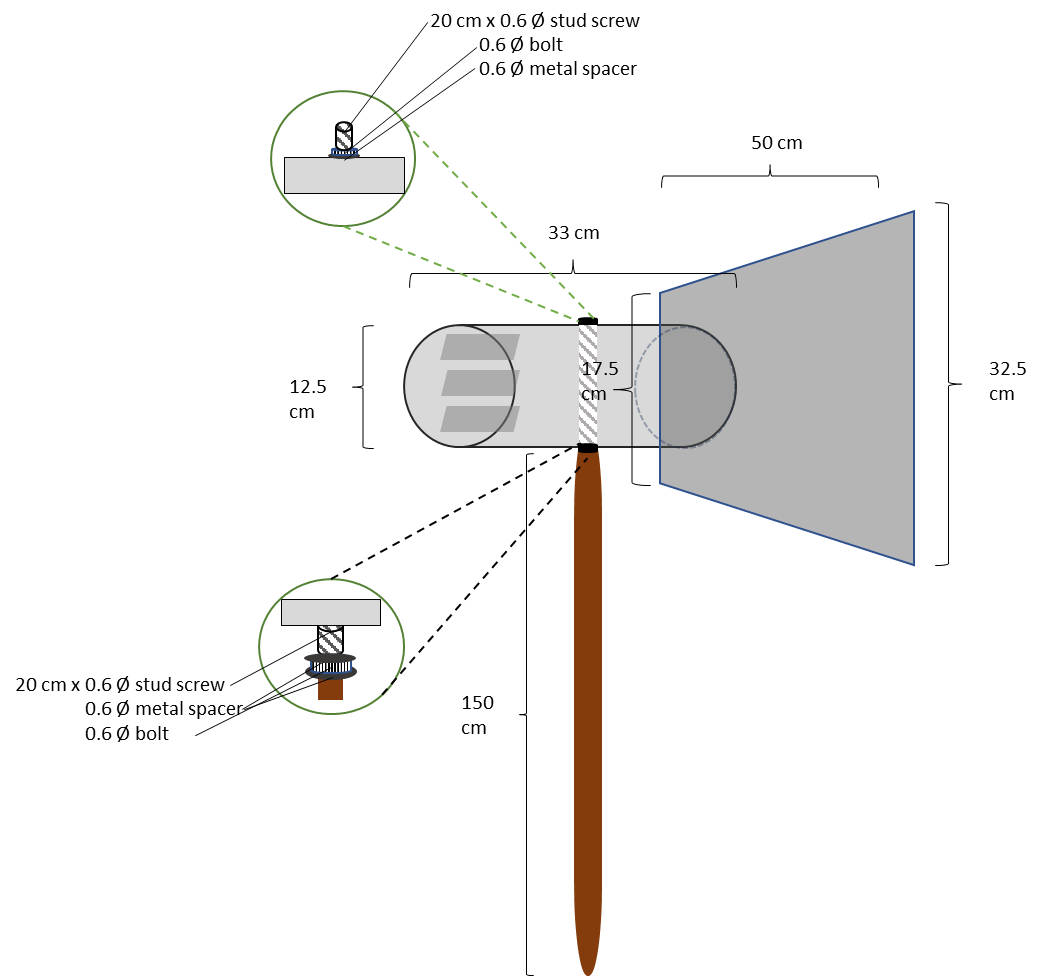


Fig. SA.3. Schematic drawing of a passive spore trap, with the dimensions of the different parts. Inside the cylinder, three microscope slides are depicted.


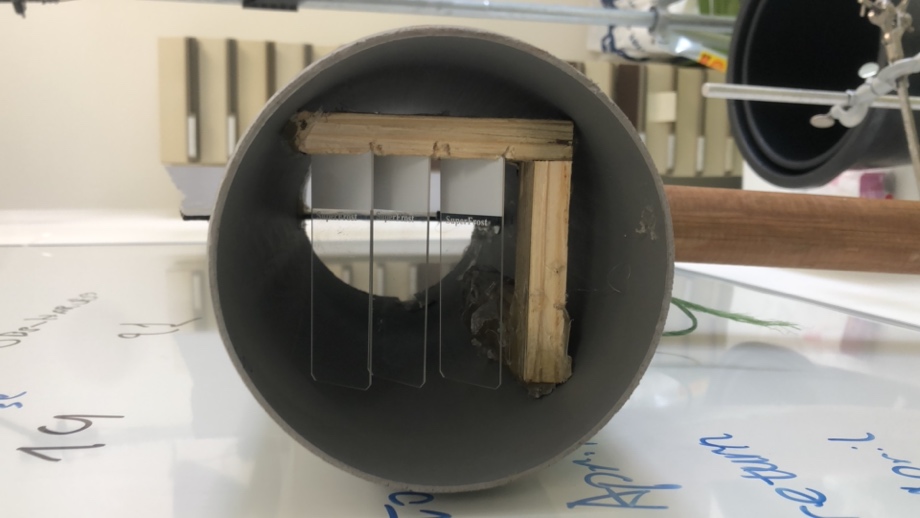


Fig. SA.4. Picture of the microscope slide holder inside the cylinder of the spore trap. The holder can hold three microscope slides at a 45 degree angle.

# Method SA.2: From wind direction to a measure of perpendicularity

Wind direction at the periphery of the field was measured every 10 minutes, returning a value between 0 and 360 degrees. This value was recalculated to a value between 0 and 1 to represent the perpendicularity of the wind in relation to the direction of the strips, using equation 1. The +20 in the equations accounts for the positioning of the strips in the 70-250 degree direction. (Fig SA.5). For example, if the wind came from a 250° direction, which is parallel to the strips, the perpendicularity would be 0. In contrast, if the wind came from a 160° direction, which is completely perpendicular to the strips, the perpendicularity would be 1. With this recalculation, wind with similar perpendicularity in relation to the strips, but coming from a different direction in degrees, would be classified similar. For example, wind coming from either a 200° or 20° direction, both has a perpendicularity of 0.77.

$$Perpendicularity=| cos \frac{\left( Wind direction degrees+20 \right)* \pi}{180} |$$

Eq. 1


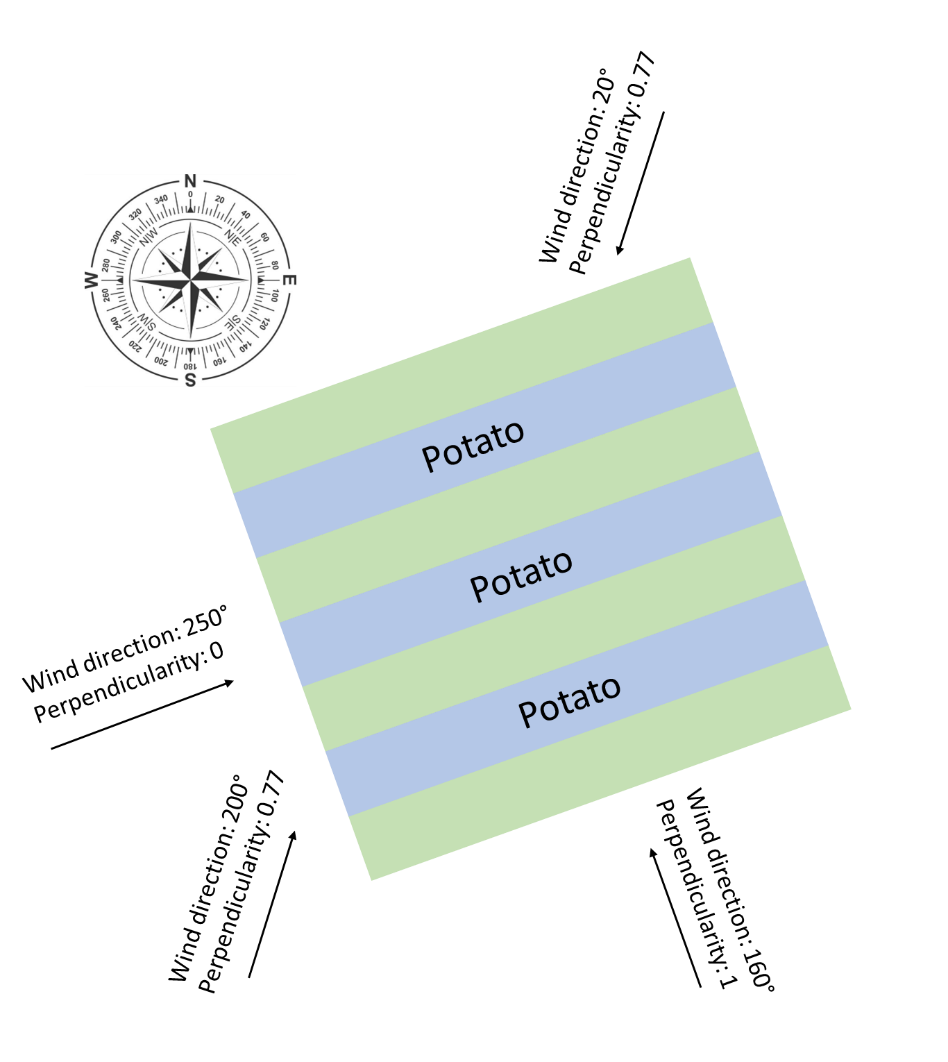


Fig. SA.5. Schematic overview of the orientation of the strips, and examples of different wind directions and their level of perpendicularity in relation to the orientation of the strips. Perpendicularity was calculated using Equation 1.


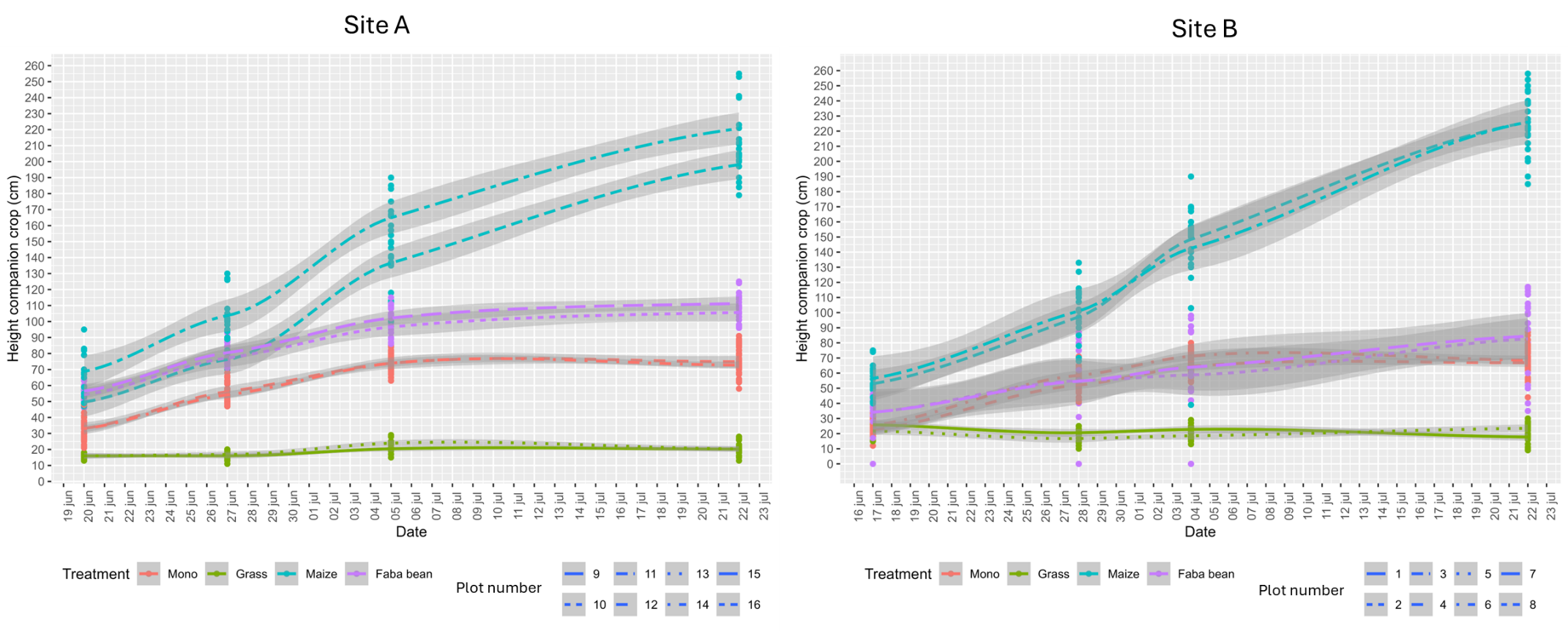


Fig. SA.6. Height of the companion crops of the strip-crop treatments with grass, maize or faba bean, at site A and B. For Potato plants grown in monoculture (mono), height of the neighbouring potato plants are presented. Points represent the measured heights, the dotted line represents the interpolation, which was used to estimate the daily height.

Table SA.1. Summary of the fitted models to the particle count data. + means additive effects are assumed, while * means main effects and interactions are estimated. A slash / before a random effect means that it is nested in the preceding random effect to the left of it.

| # | Fixed effect | Random effect | Distribution | dispformula | Log-likelihood | AIC |
| --- | --- | --- | --- | --- | --- | --- |
| 1 | Treatment * Wind Speed + Slide | Day ; Site/Trap/Slide | NB-2 | Treatment + Slide | -13323.1 | 26686.2 |
| 2 | Treatment * Wind Direction + Slide | Day ; Site/Trap/Slide | NB-2 | Treatment + Slide | -13326.7 | 26693.4 |
| 3 | Treatment + Slide | Day ; Site/Trap/Slide | NB-2 | Treatment + Slide | -13337.5 | 26707.0 |
| 4 | Treatment + Slide | Day ; Site/Trap/Slide | NB-2 | Treatment | -13341.6 | 26711.2 |
| 5 | Treatment + Slide | Day ; Site/Trap/Slide | NB-2 | Slide | -13356.1 | 26738.2 |
| 6 | Height companion * Wind Speed + Slide | Day ; Site/Trap/Slide | NB-2 | Height companion + Slide | -13356.0 | 26740.1 |
| 7 | Treatment + Slide | Day ; Site/Trap/Slide | NB-2 | - | -13360.2 | 26742.3 |
| 8 | Treatment + Slide | Day ; Site/Trap/Slide | NB-2 | Location | -13359.4 | 26742.8 |
| 9 | Height companion + Slide | Day ; Site/Trap/Slide | NB-2 | Height companion + Slide | -13365.7 | 26755.4 |
| 10 | Treatment | Day ; Site/Trap/Slide | NB-2 | - | -13381.6 | 26781.2 |
| 11 | Treatment | Day ; Site/Trap/Slide | NB-1 | - | -13603.2 | 27224.4 |
| 12 | Treatment * Wind Speed * Wind Direction + Slide | Day ; Site/Trap/Slide | NB-2 | Treatment + Slide | -13310.6 | 26677.1 |


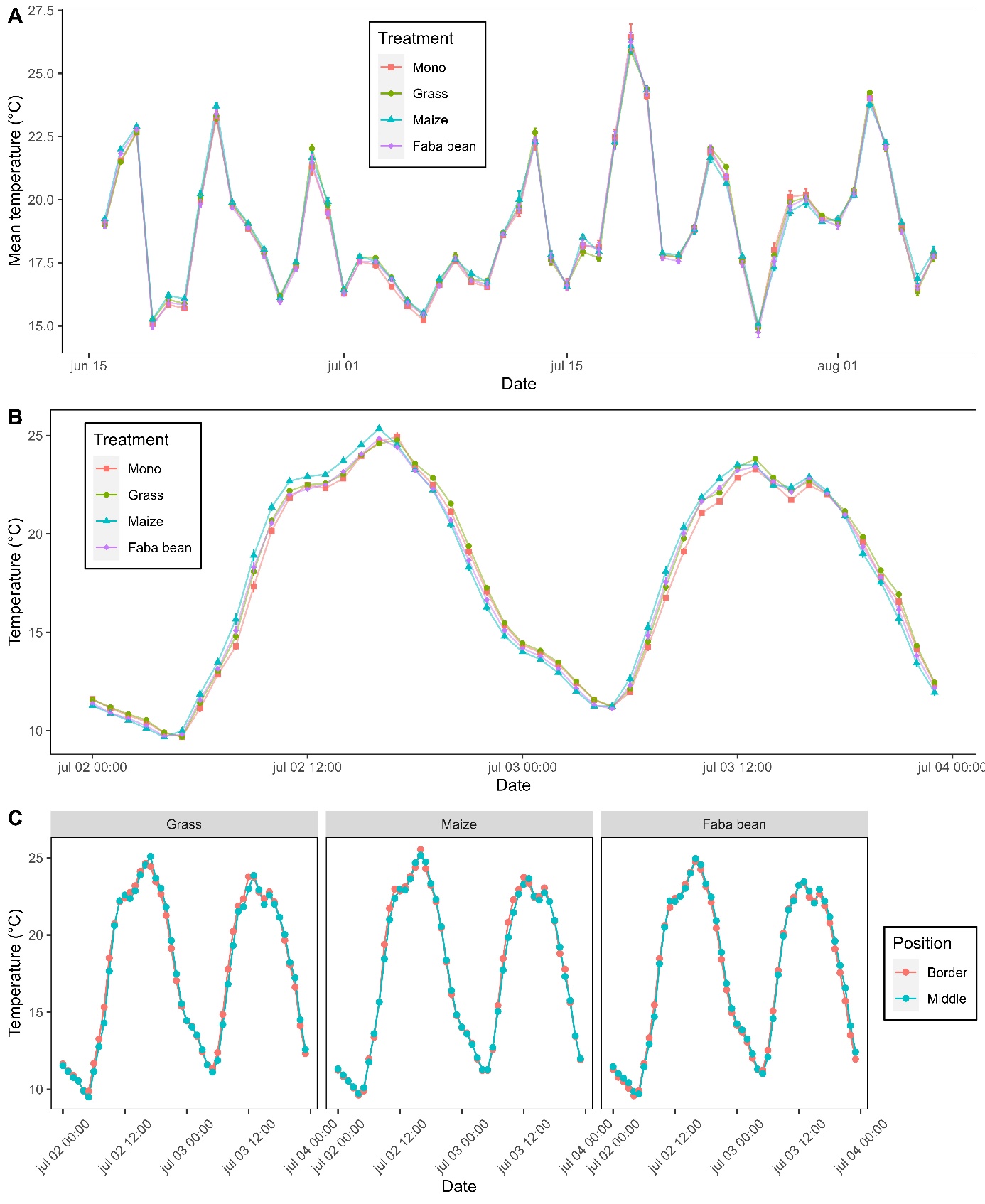


Fig. SA.7. Temperature in the potato canopy for potatoes either grown in monoculture (Mono), or strip-cropped with grass, maize or faba bean. (A) Mean daily temperature for each treatment across the growing season. (B) Hourly temperatures for each treatment between 2 and 4 July. (C) Hourly temperatures of the inner and outer rows of potato strips in the strip cropping treatments between 2 and 4 July.


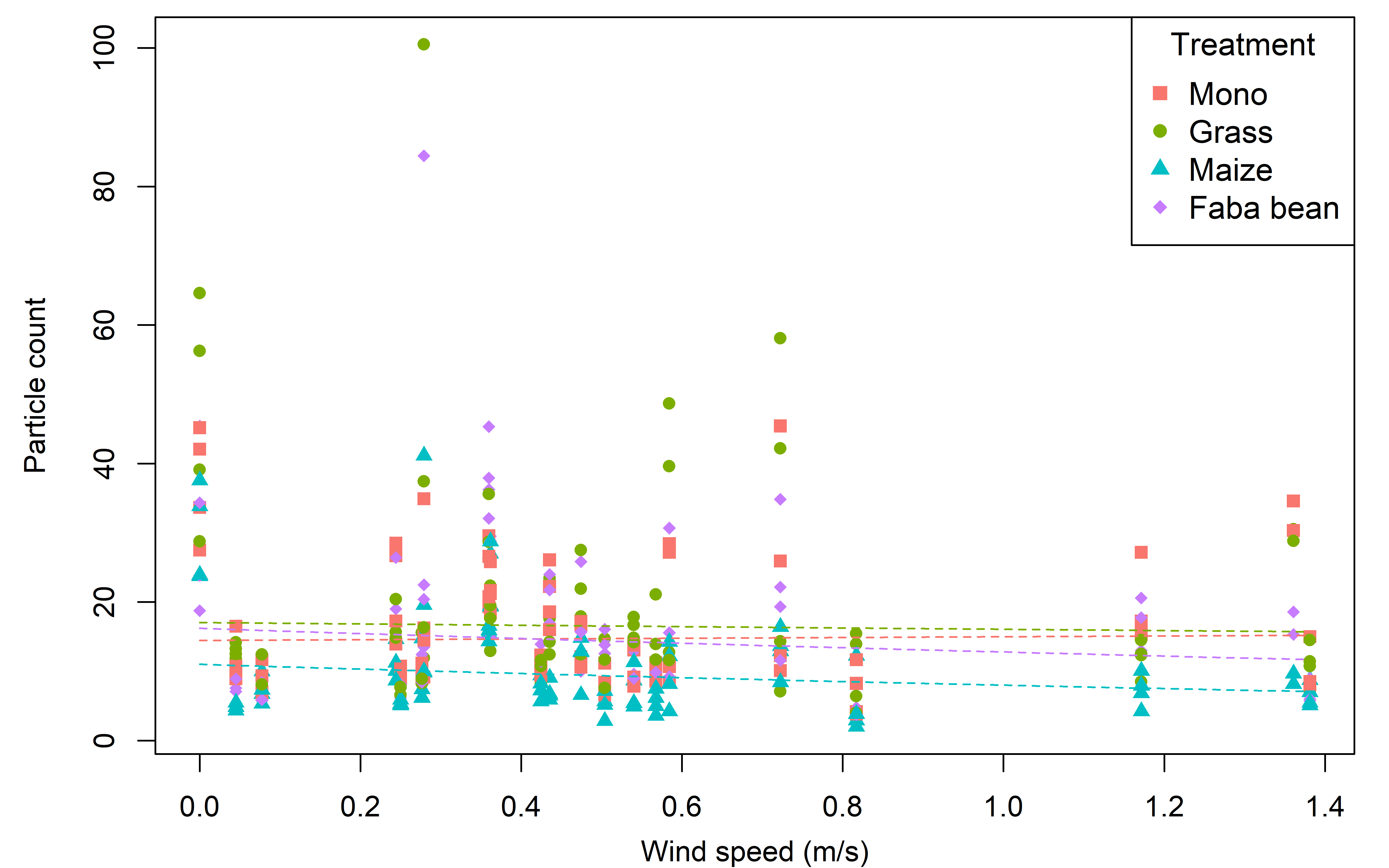


Fig. SA.8. Particle count in the potato canopy in relation to the wind speed in the 17 hours preceding collection of the microscope slides. Particles with a size between 314-1257μm2 were considered in the potato canopy for potatoes either grown in monoculture (Mono), or strip-cropped with grass, maize or faba bean. Particles were counted on 4 areas per microscope slide, each covering 19.63mm^2^. Dots represent average particle counts per spore trap per day. Increasing wind speeds were associated with significantly lower particle counts in potato-maize or potato-faba bean than the monoculture (p < 0.001 for both comparisons) or potato-grass (grass-maize: p = 0.006; grass-bean: p = 0.09).


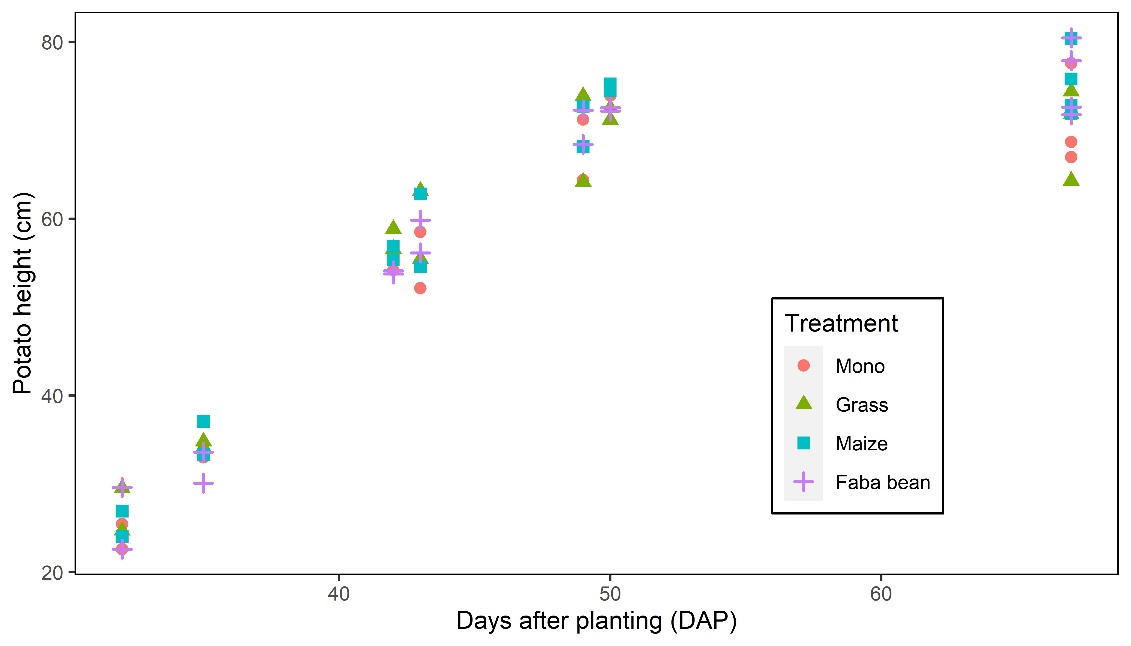


Fig. SA.9. Height of potato plants grown in monoculture (mono), or strip-cropped with either grass, maize, or faba bean across the growing season. Points represent the mean potato height per plot.

Table SA.3. Proportion of total PAR captured at four levels in the canopy of potatoes grown in monoculture or strip-cropped with grass, maize, or faba bean and its standard deviation.

|  | Canopy level | | | |
| --- | --- | --- | --- | --- |
| Treatment | Above canopy (86cm) | High canopy (58 cm) | Middle canopy (28 cm) | Low canopy (0 cm) |
| Monoculture | 0.93 ± 0.05 | 0.61 ± 0.20 | 0.19 ± 0.16 | 0.07 ± 0.05 |
| Grass | 0.90 ± 0.06 | 0.58 ± 0.22 | 0.18 ± 0.16 | 0.06 ± 0.05 |
| Maize | 0.90 ± 0.06 | 0.49 ± 0.20 | 0.12 ± 0.08 | 0.05 ± 0.04 |
| Faba bean | 0.91 ± 0.05 | 0.53 ± 0.18 | 0.14 ± 0.09 | 0.06 ± 0.05 |
