# Supplementary material for: Disease-suppressive mechanisms in contrasting potato-based strip-cropping systems
Source: Eur J Plant Pathol. 2025 May 26;173(2):469–89. doi: 10.1007/s10658-025-03073-6 (PMC12500844; doi:10.1007/s10658-025-03073-6)
Supplement: Supplementary file 2 — Supplementary file2 (DOCX 2611 KB) [file 10658_2025_3073_MOESM2_ESM.docx]

**Supplementary information B for: disease-suppressive mechanisms in contrasting potato-based strip-cropping systems**

# **Data collected in other experimental years**

Zohralyn Homulle^1^, Paola Cassiano^1^, Slava Shevchuk^1^, Niels P.R. Anten^1^, Tjeerd Jan Stomph^1^, Wopke van der Werf^1^, Jacob C. Douma^1^

^1^Centre for Crop System Analysis, Wageningen University, 6700 AK, Wageningen, the Netherlands

Corresponding author:

Zohralyn Homulle: zohralyn.homulle@wur.nl, [zohralyn@live.nl](mailto:zohralyn@live.nl)

**Table of Contents**

[**Data collected in other experimental years** 1](#_Toc196730501)

[Method SB.1: Description of the other experimental years 2](#_Toc196730502)

[Fig. SB.1. Weather conditions during the 2021, 2022 and 2024 growing season. 3](#_Toc196730503)

[**Disease severity** 4](#_Toc196730504)

[Fig. SB.2. Disease progress curves for potato late blight on potato during the 2021 and 2024 growing season, 4](#_Toc196730505)

[**Microclimate** 5](#_Toc196730506)

[Fig. SB.3. Temperature in the potato canopy for potatoes either grown in monoculture, or strip-cropped with grass, maize or faba bean. 6](#_Toc196730507)

[Fig. SB.4. Daily duration (hours) with relative humidity equal to or exceeding 90% in the potato canopy 7](#_Toc196730508)

[**Detached leaf assays** 8](#_Toc196730509)

[Fig. SB.5. Detached leaf assay of the 2021 and 2024 growing season. 9](#_Toc196730510)

[**Plant height** 10](#_Toc196730511)

[Fig. SB.6. Height of the companion crops of the strip-crop treatments with grass, maize or faba bean, in 2021 , 2022 and 2024 11](#_Toc196730512)

[Fig. SB.7. Height of potato plants grown in monoculture, or strip-cropped with either grass, maize, or faba bean during the 2021, 2022 and 2024 growing season. 12](#_Toc196730513)

# Method SB.1: Description of the other experimental years

The strip-crop experiment presented in the main paper was replicated in 2021 and 2024 using a similar setup (see Homulle et al. (2024) for details of these experiments). In 2021, the experiment had two replicate plots of potato-grass, potato-maize and potato monoculture at a single location. The 2024 setup included two replicates of sole potato, potato-grass and potato-maize, and three replicates of potato-faba bean.

In 2021, potato was planted on 28 April, and grass and maize were sown on 7 May (Fig. SB.1). In 2022, potatoes were planted later, on 17 May. Grass and maize were sown on 29 April 2022 and faba bean was sown on 3 May 2022. In 2024, potato was planted on 13 May, faba bean was sown on 21 March, grass on 29 April, and maize on 2 May.

In 2021 and 2022, first late blight symptoms were observed on 8 July. In 2024, first symptoms were observed on 10 June.

In 2021 the haulm of the potatoes was desiccated on 19 July. In 2022, the plants were desiccated on 11 August 2022. In 2024, the plants were desiccated on 9 July. In all cases, desiccation was compulsory because of the legal limit to blight severity in the field.

Below, we present the data from these additional growing seasons, including microclimate measurements, plant height, and detached leaf assay. These data allow for a broader understanding of year-to-year variability.


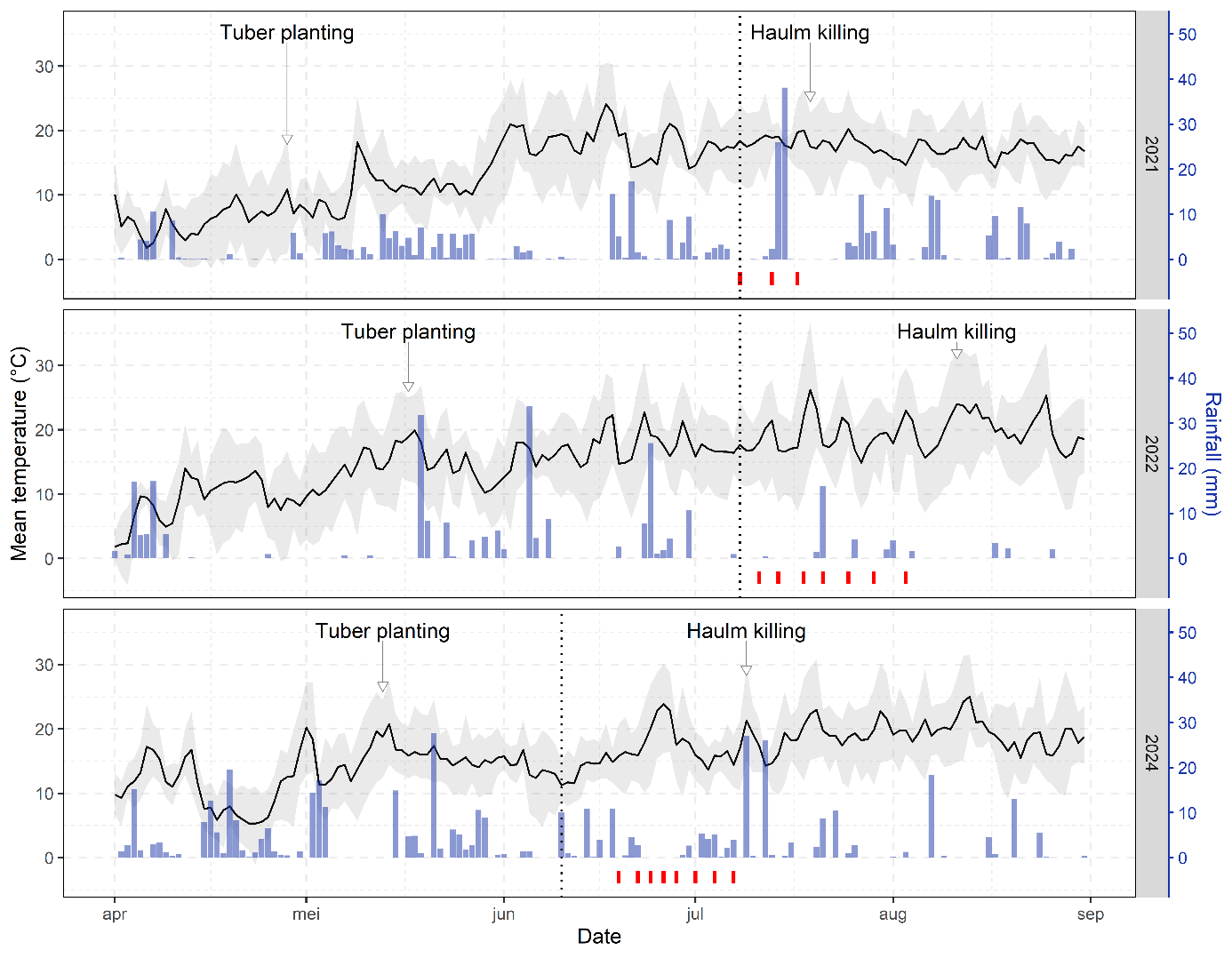


Fig. SB.1. Weather conditions during the 2021 (top), 2022 (middle) and 2024 (bottom) growing season. Red ticks on the x-axis mark late blight assessment dates, vertical dotted lines mark the first detection of late blight in each year. Black lines show mean temperature (degrees Celsius), grey ribbons span daily minimum and maximum temperatures, and blue bars are the total daily precipitation (mm). The dashes on the x-axis indicate the first of each month. Data was obtained from weather station De Veenkampen operated by Wageningen University, located approximately 3 km west of the experimental site. Adjusted from Homulle et al. (2024).

# **Disease severity**

During the 2021 growing season, wet summer conditions in the Netherlands led to rapid disease progression in potato monocultures, with average disease severity increasing from 0% to 50% in less than 10 days (Fig. SB.2A). While differences in final disease severity between treatments were not statistically significant that year, potatoes strip-cropped with grass showed had lower average disease severity (23%) compared to the monoculture (50%), with potato-maize showed intermediate values (35%).

During the 2024 growing season, the epidemic started early, with symptoms appearing only 28 days after planting. Disease suppression in potato-grass was again observed: final disease severity was 5%, compared to 12% in the monoculture (Fig. SB.2B). At the final assessment day, the potatoes strip-cropped with maize or faba bean were not significantly different from the monoculture.


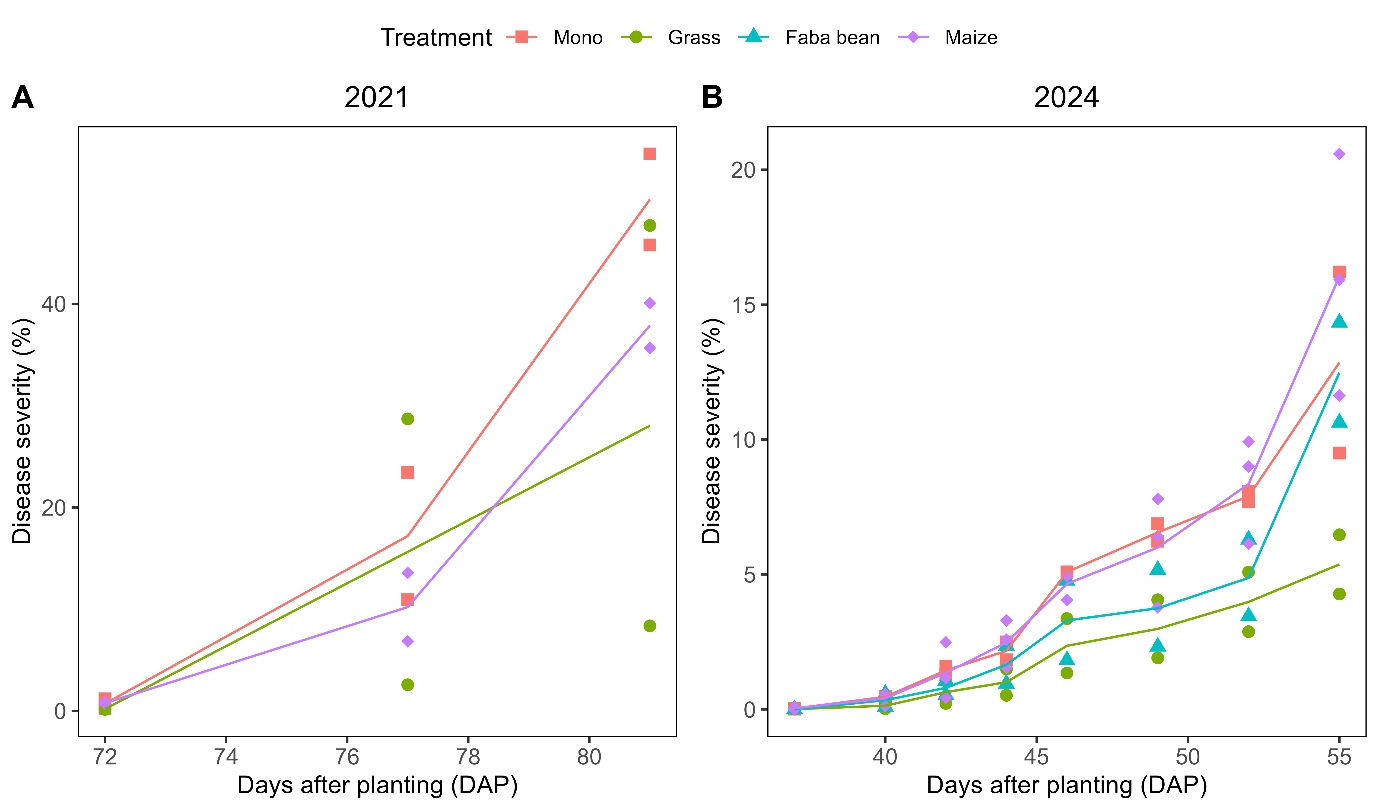


Fig. SB.2. Disease progress curves for potato late blight on potato during the 2021 (A) and 2024 (B) growing season, modified from Homulle et al. (2024). The points (symbols) represent the mean disease severity per plot based on visual observations on 36 (A) or 24 (B) plants per plot. The lines are drawn between the midpoints of the two plots for each treatment.

# **Microclimate**

In 2021, three sensors were employed per treatment. In 2024, two sensors were used in each strip-crop plot, while one sensor was used per sole potato plot.

Across the three years, temperature within the potato canopy was not systematically influenced by strip cropping, and we also found no differences among different companion crop species (Fig SB.3).

The daily duration of relative humidity above 90% was substantially lower in potato-grass than in the potato monoculture around the time of the first late blight finding in the field in both 2021 and 2022 (dotted line in Fig SB.4). This difference between potato-grass and potato mono was not observed in 2024. The 2024 potato growing season was substantially shorter than 2021 and 2022 as potato plants had to be desiccated on 9 July, which could explain why no effects were observed.


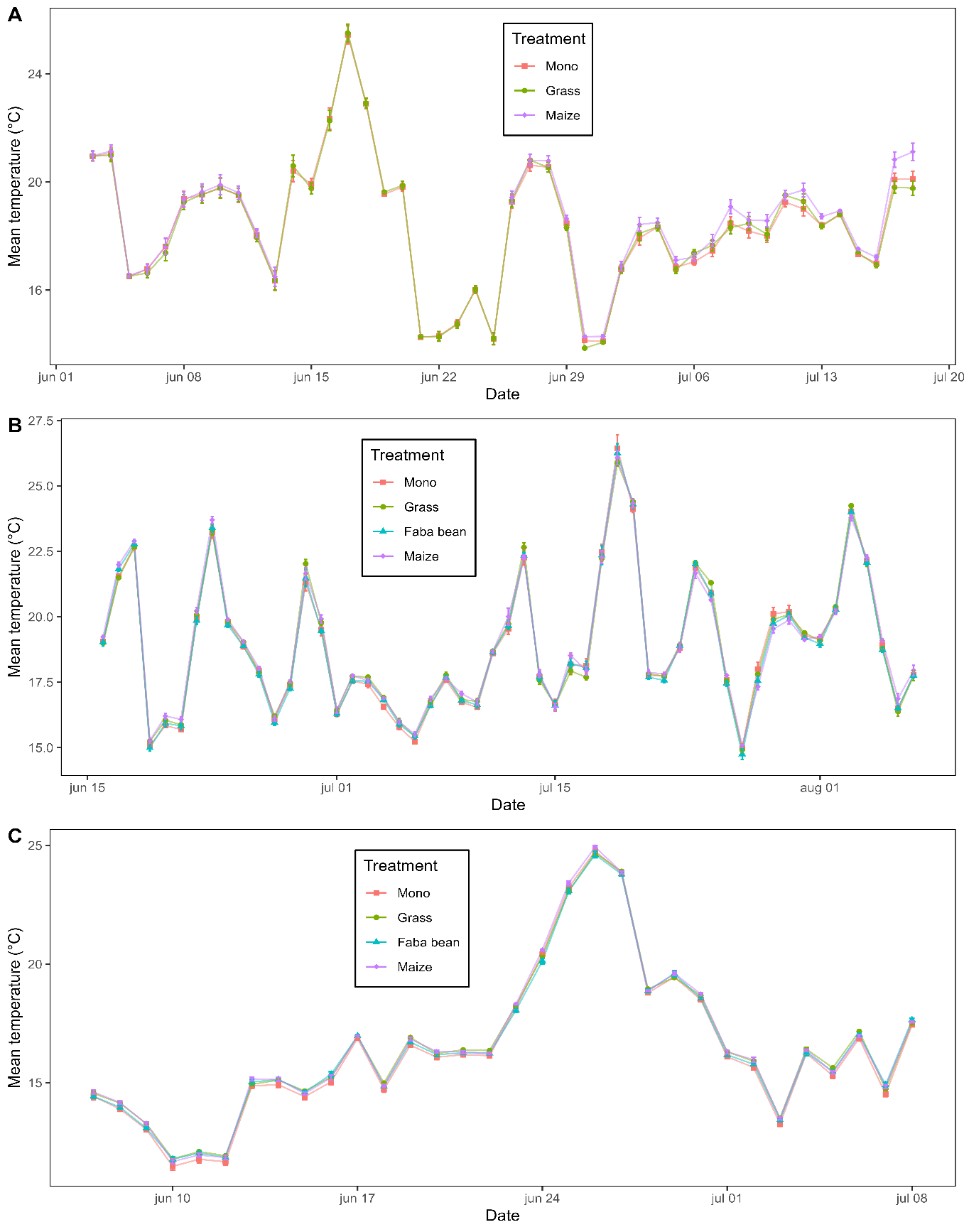


Fig. SB.3. Temperature in the potato canopy for potatoes either grown in monoculture (Mono), or strip-cropped with grass, maize or faba bean, during the 2021 (top), 2022 (middle) and 2024 (bottom) growing season.


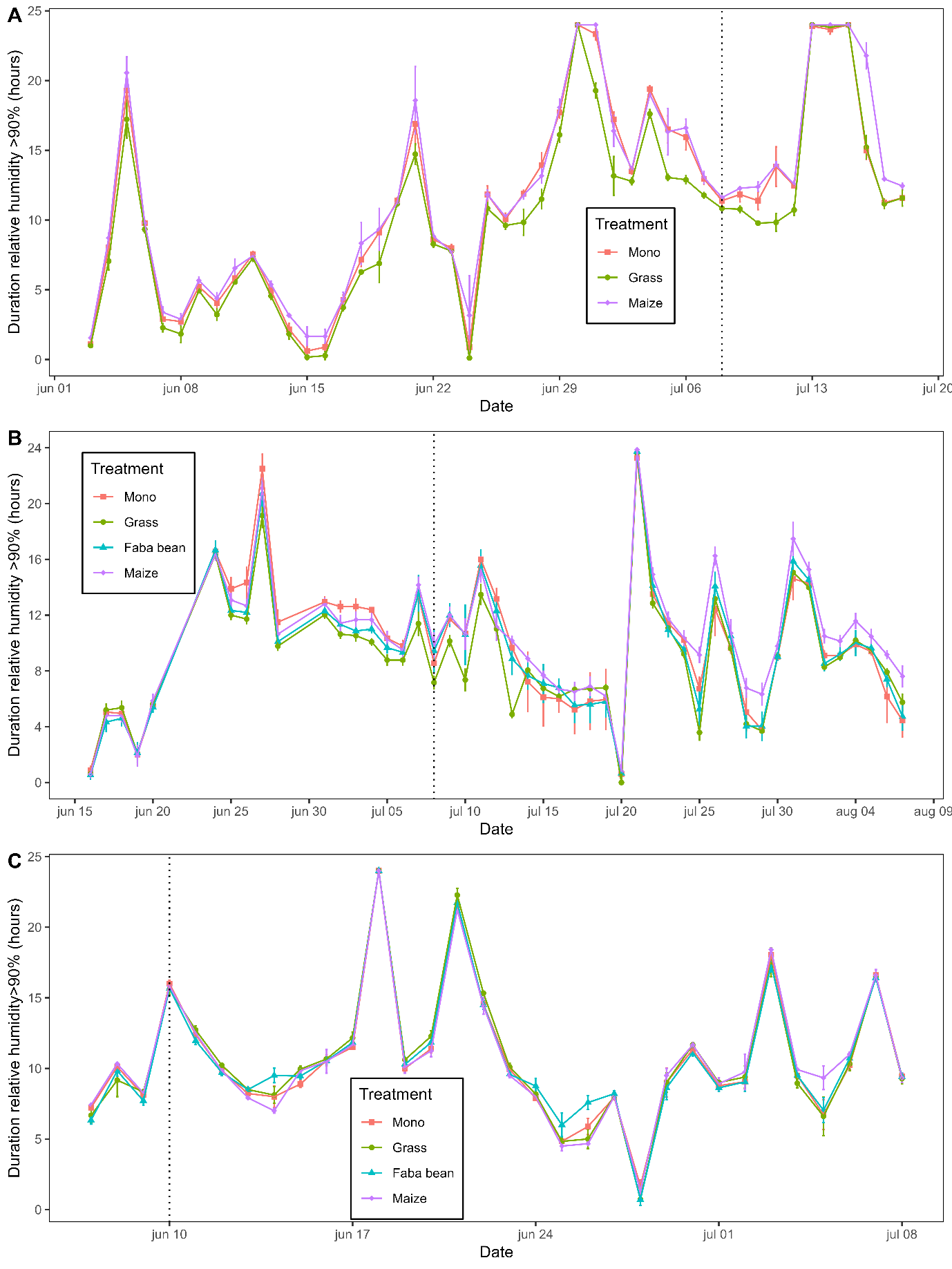


Fig. SB.4. Daily duration (hours) with relative humidity equal to or exceeding 90% in the potato canopy during the 2021 (top), 2022 (middle) and 2024 (bottom) growing season. Vertical dotted line marks the first detection of late blight. Please note: x-axis are not aligned across graphs.

# **Detached leaf assays**

Detached leaf assays were performed in 2021 and 2024.

Leaflets were collected from monocropped and strip-cropped plants at 11 weeks after potato planting in 2021. From each strip-cropping plot, ten leaflets from the inner rows and ten from both outer rows of strip-cropped potato plants were collected. Per monoculture plots, 20 leaflets were collected. Ten leaflets per strip-cropping treatment (five from the inner rows and five from the outer rows) and ten random leaflets from the monoculture plots were inoculated with distilled water only and used as a control.

In 2024, leaflets were collected from monocropped and strip-cropped plants at 5 weeks after potato planting. In each strip-cropping plot, eight leaflets were collected from the inner rows and eight from both outer potato rows. In the monoculture plots, ten leaflets were collected. Four leaflets per strip-cropping plot (two from the inner rows and two from the outer rows) and three random leaflets from the monoculture plots were inoculated with distilled water only and used as a control.

In both years, the inoculations followed the same protocol as described in the main text, using the same *P. infestans* strain and dose of sporangia.

In 2021, the leaf assay was performed when late blight was already present in the field. While the collected leaflets were symptom-free , latent infections must have been present on the leaves, as infection were found in the negative control (Fig. SB.5A). These infections interfere with the infections from the inoculations, making it challenging to assign differences between treatment. The repeat of the detached leaf assay in 2024 showed no significant differences between treatments (Fig. SB.5B).


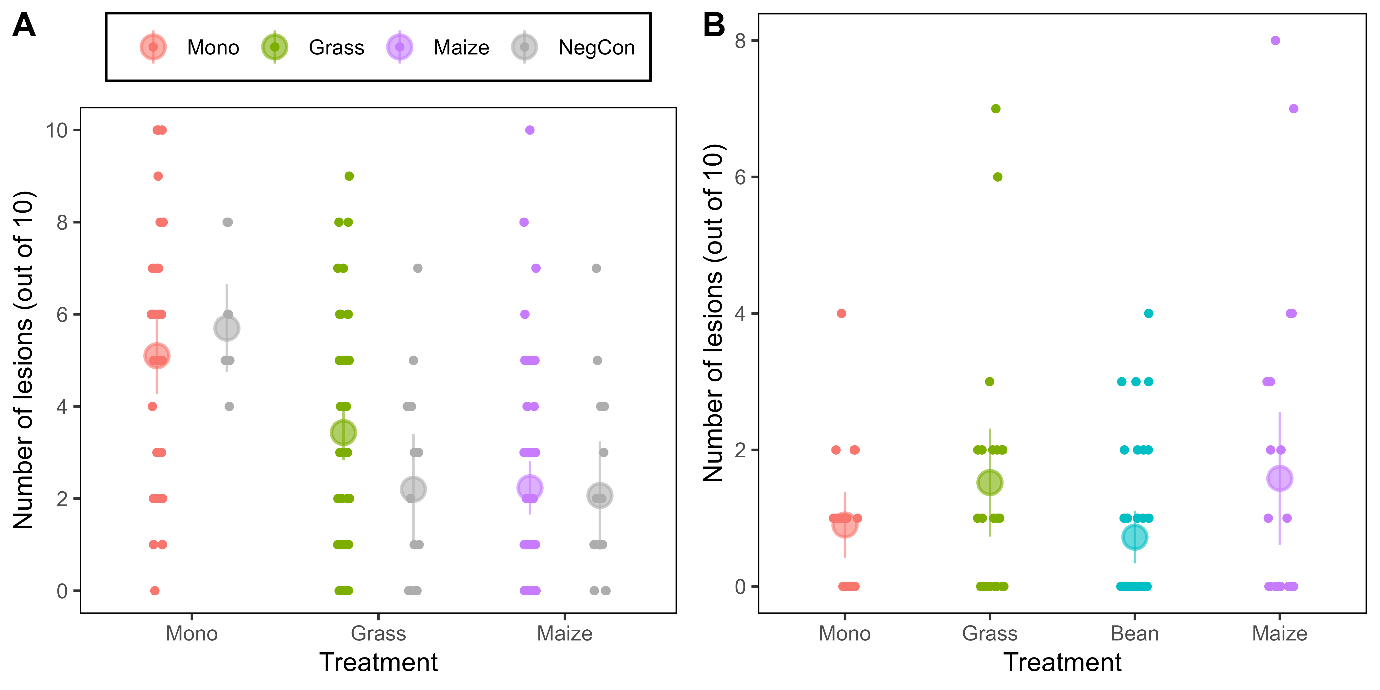


Fig. SB.5. Detached leaf assay of the 2021 (A) and 2024 (B) growing season. Number of lesions developed out of 10 droplets in detached leaf assay from potato plants grown in monoculture (mono) or strip-cropped with grass, maize, or faba bean. Grey points represent the negative control (inoculation with water). Large circles represent the means and error bars the confidence interval. The smaller points represent individual measurements.

# **Plant height**

The plant height of the potato and each companion crop species was measured in each year. We randomly selected in each plot in each of the three potato strips two transects perpendicular to the strip, with each transect comprising four plants, resulting in a total sample of 24 plants per plot. Per plot, 24 potato plants were selected from both the inner and outer rows of the strips, and their height was measured from the potato ridge until the highest point of the potato plant. The height of 12 companion plants per plot (either grass, faba bean or maize) directly neighbouring the potatoes, was also measured as the distance between the soil surface and the highest point of the plant.

heights dynamics of the companion crops differed from year to year due to differences in planting dates and weather. In 2021, maize started to surpass the potato in height around the beginning of July (Fig SB.6A). In 2022, maize was already taller than potato in mid-June, whereas faba bean was only slightly taller than potato (Fig SB.6B). In 2024, faba bean was the tallest of all the crop species for most of the growing season (Fig SB.6C). These height dynamics across time may influence the spore deposition.


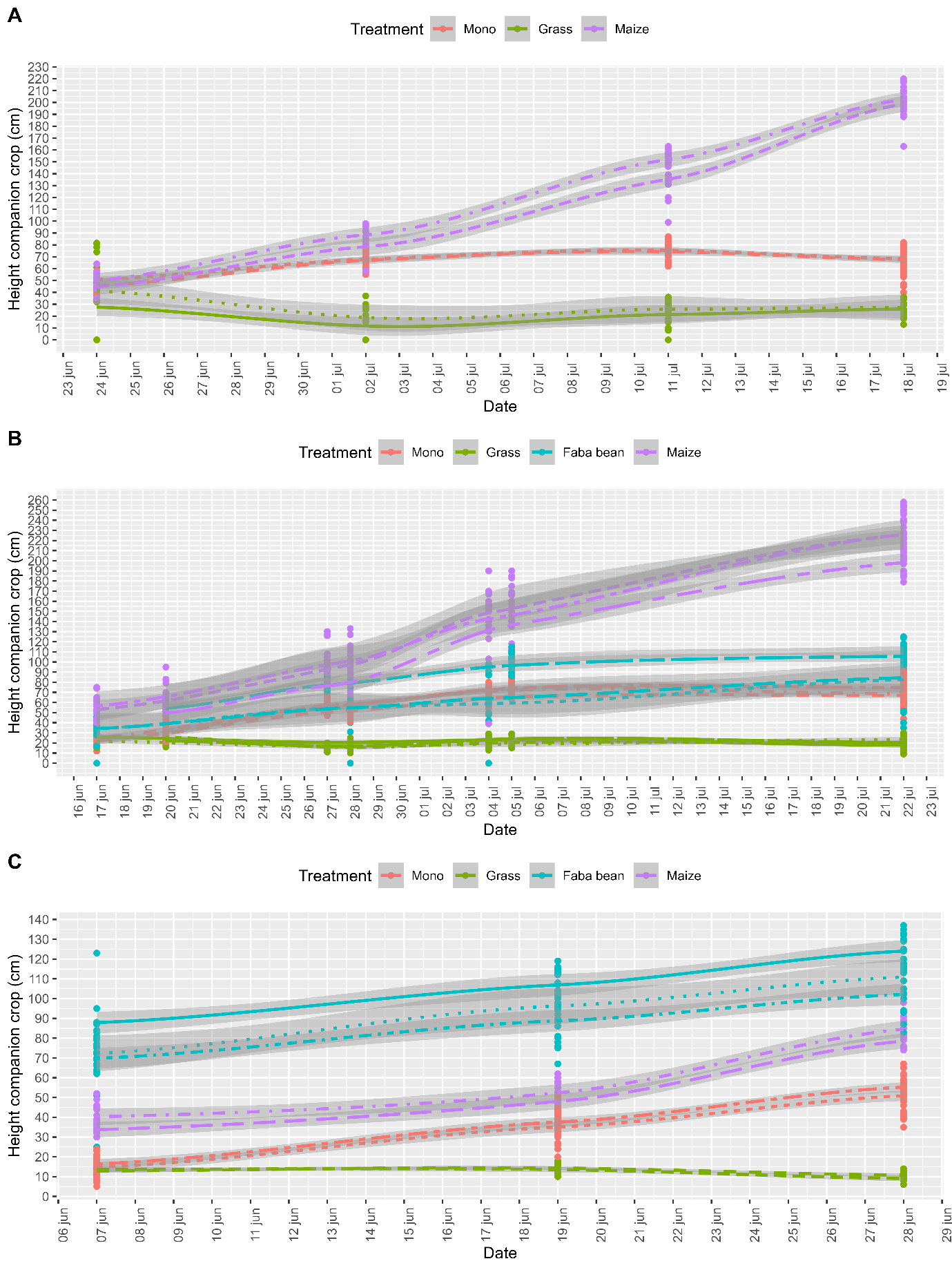


Fig. SB.6. Height of the companion crops of the strip-crop treatments with grass, maize or faba bean, in 2021 (top), 2022 (middle) and 2024 (bottom). Height of sole potato is given for comparison. For Potato plants grown in monoculture (mono), height of the neighbouring potato plants are presented. Points represent the measured heights, the dotted line is a smoothed curve through the points. Please note: x-axes differ between years.

Across the three years, the different companion crop species did not substantially influence the height of the potato plants (Fig SB.7).


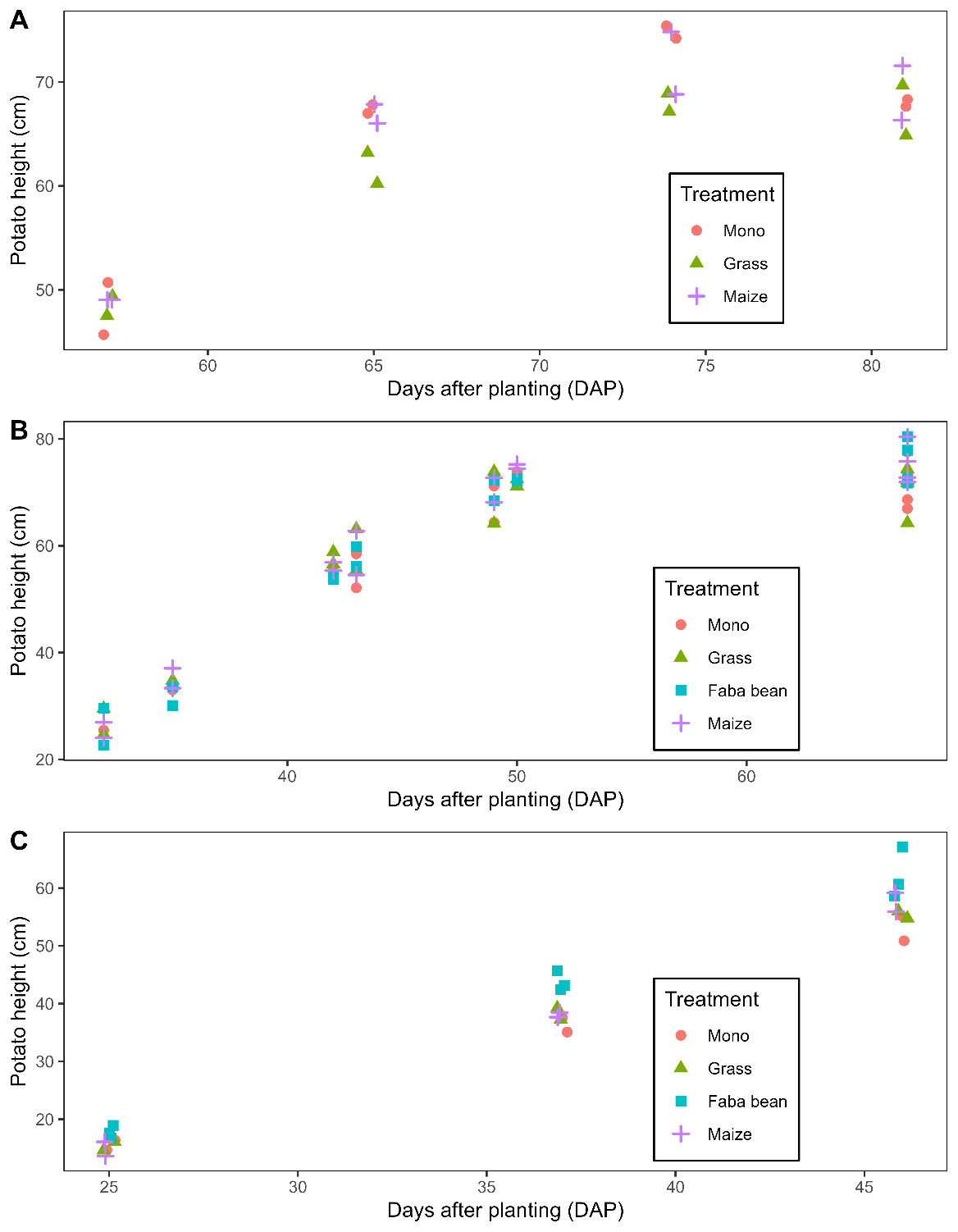


Fig. SB.7. Height of potato plants grown in monoculture (mono), or strip-cropped with either grass, maize, or faba bean during the 2021 (top), 2022 (middle) and 2024 (bottom) growing season. Points represent the mean potato height per plot. Please note: x-axis are not aligned across graphs.
